# Detailed Quasi-Classical Dynamics of the F^–^ + SiH_3_I Multi-Channel Reaction

**DOI:** 10.1021/acs.jpca.6c05884

**Published:** 2026-10-23

**Authors:** Attila Á. Dékány, Balázs J. Molnár, Gábor Czakó

**Affiliations:** MTA-SZTE Lendület “Momentum” Computational Reaction Dynamics Research Group, Interdisciplinary Excellence Centre and Department of Physical Chemistry and Materials Science, Institute of Chemistry, 37442 University of Szeged, Rerrich Béla Tér 1, Szeged H-6720, Hungary

## Abstract

We extend our previous quasi-classical trajectory study of the multi-channel F^–^ + SiH_3_I reaction on a full-dimensional analytical potential energy surface over the collision-energy range of 1–80 kcal/mol. We combine reaction probabilities and integral cross sections with initial attack-angle, scattering-angle, and product-energy distributions to characterize the major and low-probability reaction channels. Iodide substitution is the dominant reaction throughout this range, followed by proton abstraction. At low collision energies, substitution is capture dominated and strongly favors Walden inversion. At higher energies, inversion and retention cross sections become comparable, while forward scattering becomes more pronounced, particularly for inversion. Proton abstraction changes markedly with collision energy: forward scattering increases, and the product translational-energy distribution shifts toward higher energies, consistent with an increasing contribution from direct stripping. In contrast, hydride substitution and several multistep minor channels show stronger internal excitation. Taken together, these results reveal distinct collision-energy-dependent mechanisms and energy-partitioning patterns among the reaction channels of the title system.

## Graphical abstract

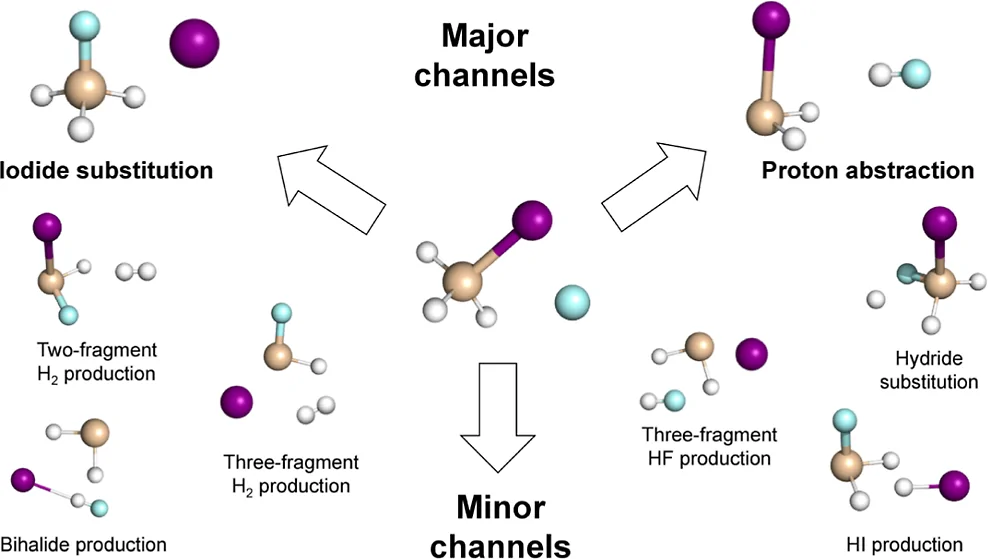

## 1 Introduction

Quasi-classical trajectory (QCT) simulations are widely used to investigate the atomistic dynamics of gas-phase reactions, providing detailed insight into their mechanisms. The reliability of these simulations depends strongly on the accuracy of the underlying energy and force calculations. In direct dynamics, energies and forces are obtained from electronic-structure calculations at every integration step. Although the direct approach is broadly applicable, its high computational cost usually limits both the number of trajectories and the level of electronic-structure theory that can be used.^1–5^ Constructing an analytical potential energy surface (PES) requires substantial initial effort, but once fitted, it allows energies and forces to be evaluated orders of magnitude faster. Consequently, hundreds of thousands or even millions of trajectories can be propagated while largely retaining the accuracy of the underlying ab initio data, within the limitations of the PES fit.^5–7^ Such large trajectory ensembles make it possible to identify and characterize low-probability reaction channels and unusual mechanisms with statistical confidence. QCT studies using fitted full-dimensional PESs, including analytical and machine-learned representations, have contributed greatly to our understanding of carbon-centered bimolecular nucleophilic substitution (S_N_2) reactions.^8–16^ In the traditional textbook model of this reaction, the nucleophile attacks from the side opposite the leaving group, and Walden inversion occurs through a nearly collinear pentacoordinate transition state.^2,17^ Modern trajectory simulations and crossed-beam experiments have revealed a more complex picture.^3,8–16,18–20^ Substitution can proceed through several alternative mechanisms and may also produce retention of configuration. In methyl halide–halide ion systems, the identified mechanisms include rebound, stripping, roundabout, front-side attack, double inversion, and various complex-mediated pathways.^3,4,16,18–20^ Their relative importance can depend on the initial conditions, such as collision energy, impact parameter, vibrational state and initial molecular orientation.^1–4,8,10,21,22^

QCT methods have also been applied to reactions with central atoms other than carbon.^23–31^ Silicon-centered systems are particularly interesting because their PESs can differ fundamentally from their carbon analogues.^29–36^ In typical carbon-centered S_N_2 reactions, the central pentacoordinate structure is a transition state, whereas the corresponding structure in silicon-centered systems is often a stable minimum.^37,38^ Reactions of fluoride with silyl halides are particularly useful for examining the effects of the central atom and the leaving group. Previous QCT studies of the F^–^ + SiH_3_Cl system showed that the reaction cannot be described by a single S_N_2 channel.^29,31^ Both inversion and retention pathways contribute significantly, alongside proton abstraction, while lower-probability channels produce HCl, H^–^, H_2_, and FHCl^–^. The F^–^ + SiH_3_I system enables direct assessment of leaving-group effects through comparison with F^–^ + SiH_3_Cl and central-atom effects through comparison with F^–^ + CH_3_I.^10^ A full-dimensional analytical PES was previously developed for the F^–^ + SiH_3_I system^30^ using the automated Robosurfer program system.^39^ The surface was represented by a permutationally invariant polynomial^7^ fitted to ManyHF-assisted^40^ composite coupled-cluster energies. Its accuracy was subsequently tested against high-level reference energies at more than eight thousand geometries sampled from QCT trajectories.^5^ The predominantly random fitting errors were comparable to or smaller than the systematic errors of lower-level methods suitable for direct dynamics. Importantly, this trajectory-based validation covered configurational regions associated with both dominant and low-probability reaction pathways, showing that the accuracy of the PES is not limited to the major product channels. The earlier PES-development study showed that the F^–^ + SiH_3_I system has an exceptionally diverse set of reaction channels. Iodide substitution and proton abstraction are the dominant processes, while lower-probability pathways analogous to those found for F^–^ + SiH_3_Cl lead to hydride substitution, HI formation, two- and three-fragment H_2_ production, and FHI^–^ formation. Both inversion and retention S_N_2 products are significant throughout the investigated collision-energy range. Although the main product channels, excitation and opacity functions, and representative mechanisms of the F^–^ + SiH_3_I reaction have already been reported,^5,30^ several aspects of its dynamics remain unexplored. We extend this investigation by reanalyzing the generated QCT ensemble. The previously reported integral cross sections and opacity functions, including the separation of the S_N_2 channel into inversion and retention contributions, are included to provide context for the present work. Initial attack-angle, scattering-angle, and product translational, rotational, and vibrational energy distributions are then used to examine the effects of collision energy, impact parameter, and initial orientation. Comparisons with the F^–^ + SiH_3_Cl and F^–^ + CH_3_I systems are used to assess the effects of replacing Cl by I and C by Si, respectively.

## 2 Methods and Computational Details

The present study provides a more detailed analysis of the QCT ensemble generated in our previous investigation^30^ of the F^–^ + SiH_3_I reaction. No additional trajectories were propagated. Instead, the existing trajectory data set was used to obtain additional trajectory-level observables, including initial attack-angle and scattering-angle distributions, as well as product energy distributions. The PES was generated using an iterative process, automated by the Robosurfer program system. The initial fitting data set was constructed by random perturbations of stationary-point geometries and contained slightly more than 10,000 energy points. The ab initio energies were computed at the ManyHF-[CCSD-F12b + BCCD­(T) – BCCD]/aug-cc-pVTZ­(-PP) composite coupled-cluster level using MOLPRO 2015.1.^41–43^ The aug-cc-pVTZ­(-PP) notation means that for the lighter atoms (H, F, Si) the augmented correlation-consistent triple-ζ basis set (aug-cc-pVTZ) was employed, whereas for the iodine atom a relativistic effective core potential (ECP) was used with the corresponding aug-cc-pVTZ-PP basis set.^44–47^ The combination is referred to as AVTZ­(-PP) throughout this work. The Brueckner-type perturbative triples correction, evaluated as *E*_BCCD(T)_–*E*_BCCD_, was employed because of the instability of the (T) term of the conventional CCSD­(T) method,^48,49^ and convergence issues in the underlying Hartree–Fock computations were mitigated using the ManyHF approach.^40^ The PES is represented as a permutationally invariant polynomial in Morse-variables.^7,50^ The final fitting set of the PES contains 19,492 energy points and achieves chemical accuracy in the energetically relevant regions.^30^

The initial vibrational energy for each trajectory was set to the zero-point vibrational energy (ZPVE) of the system via normal-mode sampling,^51^ matching the ZPVE of the polyatomic reactant, SiH_3_I. The rotational energy was set to zero. Vibrational phases and the relative orientations of the reactants were generated using a Monte Carlo sampling.

For each trajectory, the collision energy (*E*_coll_) and impact parameter (*b*) were fixed. The collision energy is defined as the relative translational energy of the reactants in the center-of-mass frame, while the impact parameter is given by *b* = |**r** − (**v**·**r**)**v**|, where **r** is the vector connecting the centers of mass of the two reactant fragments and **v** is the unit vector along the initial relative velocity of the reactants.

The initial center-of-mass separation of the reactants was defined as $\sqrt{x^{2}+b^{2}}$ , with *x* = 40 bohr at 1 kcal/mol collision energy and *x* = 25 bohr at higher collision energies, ensuring negligible initial interaction between F^–^ and SiH_3_I.

For each (*E*_coll_, *b*) pair, 5000 independent trajectories were propagated. The collision energy range spanned 1–80 kcal/mol. Impact parameters were sampled from 0 to *b*_max_ in 0.5 bohr increments, where *b*_max_ is defined as the smallest impact parameter for which only nonreactive trajectories are observed. The corresponding *b*_max_ values decrease with increasing collision energy, from 26.5 bohr at *E*_coll_ = 1 kcal/mol to 11 bohr at 80 kcal/mol collision energy.

The classical equations of motion were integrated using an in-house Fortran code employing the velocity-Verlet algorithm^52,53^ with a fixed time step of 3 atomic units (∼72.57 attoseconds). Trajectories were terminated when the largest interatomic distance exceeded the maximum initial interatomic distance by 1 bohr, ensuring that the product fragments are well separated.

Product channels were identified based on the atomic connectivity in the final step of each trajectory. A product structure was considered successfully assigned if its adjacency list matched one of the permutationally invariant adjacency lists corresponding to predefined reference structures. In the title system, all examined product channels have different connectivity patterns, making the distinction straightforward. In highly distorted geometries, the instantaneous connectivity may not correspond to any reference structure, resulting in unidentified products. In the case of two coproducts, the large separation and opposite motion of the fragments at the termination step ensure that further interaction would only decrease upon continued propagation. In contrast, for three-fragment configurations, only one fragment can be guaranteed to be sufficiently separated, while the remaining two fragments may still interact. Consequently, the trajectory may be terminated before the completion of a multistep reaction, or a highly elongated vibrational motion may be misinterpreted as bond breaking.

To reduce misidentification, trajectories with ambiguous final configurations were propagated for several thousand additional integration steps. The final configuration was replaced if a more reliable assignment could be obtained from the extended trajectory. For initially unidentifiable structures, the first configuration along the extended trajectory that could be assigned to a reference structure was selected. In the case of three-fragment configurations, preference was given to the first configuration corresponding to a two-fragment separation. If an unidentifiable structure was followed by identifiable three-fragment and subsequently two-fragment configurations, the first two-fragment configuration was used for final product assignment.

The stereochemical outcome (inversion or retention) was determined using a vector projection-based ligand orientation descriptor. This method quantifies the orientation of the leaving group relative to the central atom by projecting the ligand–central atom vector onto a predefined reference axis associated with the molecular framework. A change in the sign of this projection between the reactant and S_N_2 product configurations corresponds to inversion, whereas preservation of the sign indicates retention. This approach enables a robust classification of stereochemical pathways even in highly distorted geometries and has been successfully applied in previous studies of S_N_2 reaction dynamics.^54^

The reaction probability (*P*) for a given collision energy and impact parameter is defined as

1 $$P\left(E_{coll},b\right)=\frac{N_{react}\left(E_{coll},b\right)}{N}$$

where *N*_react_ is the number of trajectories leading to a given product formation and *N* is the total number of trajectories for each (*E*_coll_,*b*) bin (in our case, *N* = 5000). The integral cross section (ICS, or σ) for a given product channel is calculated by integrating the reaction probability over the impact parameter at a fixed collision energy.

2 $$\sigma \left(E_{coll}\right)=2\pi \int_{b=0}^{b_{\max}}P\left({b;E}_{coll}\right)bdb$$

We obtained the ICS values via numerical integration on the discrete impact parameter grid using the trapezoidal rule.

Three types of ICSs were calculated: vibrationally unconstrained, soft-constrained, and hard-constrained. For all three, the denominator is the total number of trajectories launched in each (*E*_coll_, *b*) bin. For the unconstrained ICS, the numerator includes all trajectories assigned to the product channel under consideration. The soft constraint requires that the total product vibrational energy, summed over all product fragments, be at least equal to the corresponding total zero-point vibrational energy. The hard constraint is stricter, as it requires that each product fragment individually satisfy its ZPVE condition.

The statistical uncertainty of the reaction probabilities was estimated assuming independent Bernoulli trials within each (*E*_coll_, *b*) bin. The standard error (SE) is given by

$$SE\left[P\left(b\right)\right]=\sqrt{\frac{P\left(b\right)\left[1-P\left(b\right)\right]}{N}}$$ 3

The corresponding integral cross-section standard error was obtained as

4 $$SE\left(\sigma \right)=\sqrt{\sum_{i=1}^{n}{\left(2\pi b_{i}w_{i}SE\left[P\left(b_{i}\right)\right]\right)}^{2}}$$

where *b*_*i*_ is the *i*-th sampled impact parameter, and *w*_*i*_ are the trapezoidal integration weights, with *w*_1_ = *w*_*n*_ = Δ*b*/2, and *w*_*i*_ = Δ*b* (1 < *i* < *n*).

The initial attack angle (α) is defined as the angle between the center-of-mass velocity vector of a selected reactant fragment and the vector pointing from its central atom to a selected directly bonded ligand, as illustrated in Figure 1. Although the choice of ligand is conventional, I is the natural choice for SiH_3_I because the remaining three ligands are symmetry-equivalent hydrogen atoms. The distributions are expressed in terms of cos­(α), for which uniformity indicates no preferred attack orientation. Back-side attack corresponds to cos­(α) ≈ –1 (α ≈ 180°) and front-side attack means cos­(α) ≈ 1 (α ≈ 0°).

**Figure 1:**
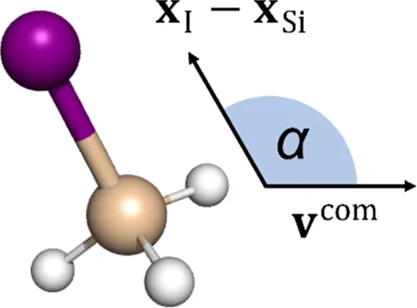
Definition of the initial attack angle for the F^–^ + SiH_3_I reaction. The angle α is defined as the angle between the center-of-mass velocity vector of SiH_3_I and the Si–I bond vector at the initial geometry.

The scattering angle (θ) is defined as the angle between the initial center-of-mass velocity vector of the F^–^ reactant and the center-of-mass velocity vector of a selected product fragment. The selection depends on the product channel. In the case of the formation of two coproducts, we follow the fluorine-containing fragment, and for three-fragment product channels, we follow the anionic species. The scattering angle distributions are computed in terms of cos­(θ). Forward scattering corresponds to cos­(θ) ≈ 1, and backward scattering refers to large deflection angles, cos­(θ) ≈ –1. A flat distribution in cos­(θ) indicates no preferred scattering direction.

The postreaction energy of the products is decomposed into translational, rotational, vibrational, and internal energy components using classical expressions. The total internal energy is defined as the sum of vibrational and rotational contributions: *E*_int_ = *E*_vib_ + *E*_rot_. These quantities are evaluated for each trajectory and used to construct energy distributions. These energy distributions provide insight into the directness of the reaction mechanisms. In direct reactions, a significant fraction of the collision energy is converted into product translation, whereas in indirect, complex-forming mechanisms the energy is redistributed into internal degrees of freedom, such as vibration and rotation. Angular and product-energy distributions were calculated using all trajectories assigned to the corresponding product channel, without applying vibrational energy constraints.

Differential cross sections (DCSs) describe the distribution of the integral cross section with respect to a trajectory-level observable. The observable *A* is divided into equal-width intervals *A*_*k*_ of width Δ*A*. For each collision energy and impact parameter, the probability associated with the *k*-th interval is

5 $$P\left(A_{k},b;E_{coll}\right)=\frac{N_{react}\left(A_{k},b;E_{coll}\right)}{N}$$

here, *N*_react_(*A*_*k*_, *b*; *E*_coll_) is the number of trajectories that are assigned to a given product channel and have an observable value within *A*_*k*_. The corresponding contribution to the cross section is

6 $${\Delta \sigma }_{k}\left(E_{coll}\right)=2\pi \int_{0}^{b_{\max}}P\left(A_{k},b;E_{coll}\right)bdb$$

The integral is evaluated numerically over the discrete impact-parameter grid using the trapezoidal rule. Thus, Δσ_*k*_ is the cross-section contribution from trajectories whose observable values lie within *A*_*k*_. The bin-averaged differential cross section is obtained by dividing the cross-section contribution by the bin width

7 $${\left(\frac{d\sigma }{dA}\right)}_{k}=\frac{{\Delta \sigma }_{k}\left(E_{coll}\right)}{\Delta A}=\frac{2\pi }{\Delta A}\int_{b=0}^{b_{\max}}P\left(A_{k},b;E_{coll}\right)bdb$$

The normalized distribution is

8 $$f_{k}\left(E_{coll}\right)=\frac{1}{\sigma \left(E_{coll}\right)}{\left(\frac{d\sigma }{dA}\right)}_{k}$$

This distribution satisfies $\sum_{k}f_{k}\left(E_{coll}\right)\Delta A=1$ , provided that the binning range includes all values of the observable.

The postprocessing and analysis of the trajectory data were carried out using the open-source Python packages qcta^55^ and qctresults,^56^ which were developed in our research group. The qcta package provides tools for trajectory assignments and for computing molecular properties such as energies, center-of-mass velocities, and ligand orientation descriptors. The qctresults package was used to extract per-trajectory observables, perform trajectory filtering (e.g., by product channel, vibrational constraints or stereochemistry for the S_N_2 trajectories), and compute statistical quantities, including reaction probabilities, integral cross sections, and differential cross sections via binning and numerical integration over the impact parameter.

## 3 Results and Discussion

### 3.1 Potential Energy Surface

Figure 2 shows the schematic potential energy surface (PES) of the gas-phase SiH_3_I + F^–^ system and the relative energies of its characteristic stationary points. No transition states are involved in any of the product-forming pathways. All energies are relative to the energy of the reactant structure optimized on the PES. Values outside parentheses correspond to classical energies, representing electronic energies evaluated at the stationary-point geometries without zero-point vibrational contributions. Values in parentheses represent adiabatic energies, obtained by adding harmonic zero-point vibrational energy (ZPVE) corrections to the classical energies.

**Figure 2:**
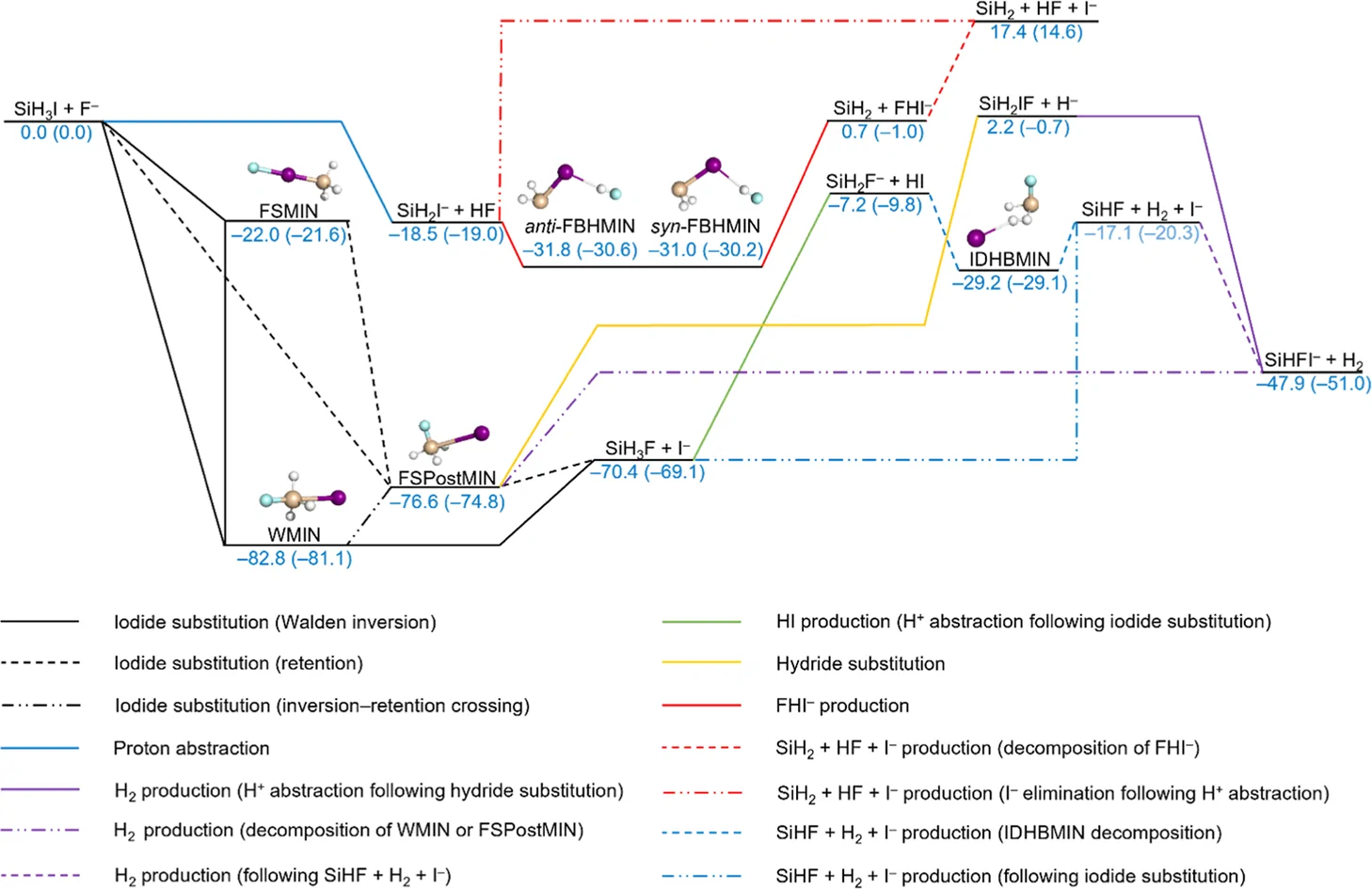
Schematic potential energy surface of the F^–^ + SiH_3_I gas-phase reaction. The scheme summarizes the product-forming pathways. Some product channels are reached through multistep mechanisms. Intermediate minima are represented by molecular structures. Energies (in kcal/mol) are given relative to the PES optimized reactants and correspond to classical (adiabatic) values obtained from the analytical PES.^30^ Adapted with permission from ref 30. Copyright 2024 AIP Publishing.

The PES describes multiple product formations, including iodide substitution yielding SiH_3_F + I^–^ through both inversion and retention channels. These reactions are characterized by single-well potentials featuring deep negative-energy minima, with WMIN for the Walden inversion and FSPostMIN for the retention channel. The low energies of these complexes make both pathways accessible even at low collision energies. This behavior contrasts with the F^–^ + CH_3_I system, where Walden inversion proceeds on a double-well potential and direct front-side retention involves a positive-energy barrier, while the lower-energy retention channel is double inversion.^10^ The energetic accessibility of both stereochemical outcomes resembles that of F^–^ + SiH_3_Cl, although inversion strongly dominates the low-energy dynamics of the silyl chloride system.^29,31^ The front-side route of the chloride PES nevertheless exhibits a submerged FSPreMIN–FSTS–FSPostMIN double-well profile, whereas the present PES contains only the deep FSPostMIN well.^29^ Since analogous double-well profiles are also found for the schematic PESs of Cl^–^ + SiH_3_I and Br^–^ + SiH_3_I, the single-well for the F^–^ + SiH_3_I system appears atypical.^38^ WMIN and FSPostMIN can also interconvert, enabling trajectories to switch between inversion and retention. Both WMIN and FSPostMIN can also decompose via hydride-ion substitution, yielding SiH_2_FI + H^–^. This reaction is nearly thermoneutral in the F^–^ + SiH_3_Cl and F^–^ + SiH_3_I systems, but highly endothermic in F^–^ + CH_3_I, demonstrating a pronounced central-atom effect.^29,38^ The proton abstraction producing SiH_2_I^–^ + HF is a barrierless, exothermic reaction. The analogous reaction is endothermic in F^–^ + CH_3_I and significantly less exothermic in F^–^ + SiH_3_Cl, showing that both replacement of C by Si and replacement of Cl by I make proton abstraction thermochemically more favorable.^29,38^ Hydride- and iodide-ion substitutions can also be followed by proton abstractions performed by the leaving ion, yielding SiHFI^–^ + H_2_ and SiH_2_F^–^ + HI, respectively. Additional channels include halogen-ion abstraction following the formation of SiH_2_I^–^ + HF and FBHMIN intermediates (*syn*- and *anti*-conformers) to yield SiH_2_ + FHI^–^. The SiH_2_ + FHCl^–^ channel of the F^–^ + SiH_3_Cl reaction is endothermic and appears only at higher collision energies with low probability, whereas the FHI^–^ formation of the title system is nearly thermoneutral,^29,31^ demonstrating that replacement of Cl by I makes bihalide formation energetically more favorable. At sufficiently high energy, FHI^–^ can dissociate further, yielding the three-fragment products SiH_2_ + HF + I^–^. The analogous SiH_2_ + HF + Cl^–^ channel is substantially more endothermic.^29^ Besides H_2_ formation via proton abstraction of the hydride ion, molecular hydrogen can also be generated through a multistep mechanism via SiH_2_F^–^ + HI production, the subsequent formation of a dihydrogen-bonded complex (IDHBMIN), and the dissociation of this complex to SiHF + H_2_ + I^–^. Subsequent recombination of SiHF with I^–^ provides an alternative pathway back to the two-fragment products, SiHFI^–^ + H_2_. An analogous ClDHBMIN-mediated mechanism occurs in the F^–^ + SiH_3_Cl system as well,^29,31^ whereas comparable dihydrogen-bonded complexes have not been reported for carbon-centered ion–molecule reactions.

A halogen-bonded entrance-channel minimum, denoted FSMIN, was also identified. It is included in the schematic, but the subsequent SiH_3_ + FI^–^ halogen abstraction pathway is omitted because this region is not reliably represented by the present PES. A complex analogous to FSMIN occurs in the F^–^ + CH_3_I system, where it substantially affects the dynamics, whereas no corresponding F–Cl halogen-bonded minimum was found for F^–^ + SiH_3_Cl.^10,29^

Overall, the present PES combines characteristic features of silicon-centered substitution with pronounced iodine-specific effects. The silicon center gives rise to deeply submerged inversion and retention pathways and facilitates several secondary reactions, while replacement of Cl by I makes substitution and proton abstraction more exothermic, lowers the energies of the bihalide and three-fragment product channels, and enables the formation of a halogen-bonded entrance-channel complex.

### 3.2 Reaction Probabilities and Integral Cross Sections

Figures 3, 4, 5 and 6 present the reaction probabilities and integral cross sections for the reactions of the SiH_3_I + F^–^ system. ICS values are presented with hard and soft vibrational energy constraints, as well as without energy constraints. Shaded regions indicate ±1 standard error for the ICSs. For the major channels, the relative standard errors remain below 1.2%. For the minor channels, the absolute standard errors are less than 0.17 bohr,^2^ but the relative uncertainties are typically 5–20%. Substantially larger relative uncertainties occur at extremely small cross sections. To avoid visual clutter, error bands are omitted from the reaction-probability plots in Figures 3 and 4. Reaction-probability plots with standard-error bands at selected collision energies are provided in the Supporting Information as Figures S1 and S2.

**Figure 3:**
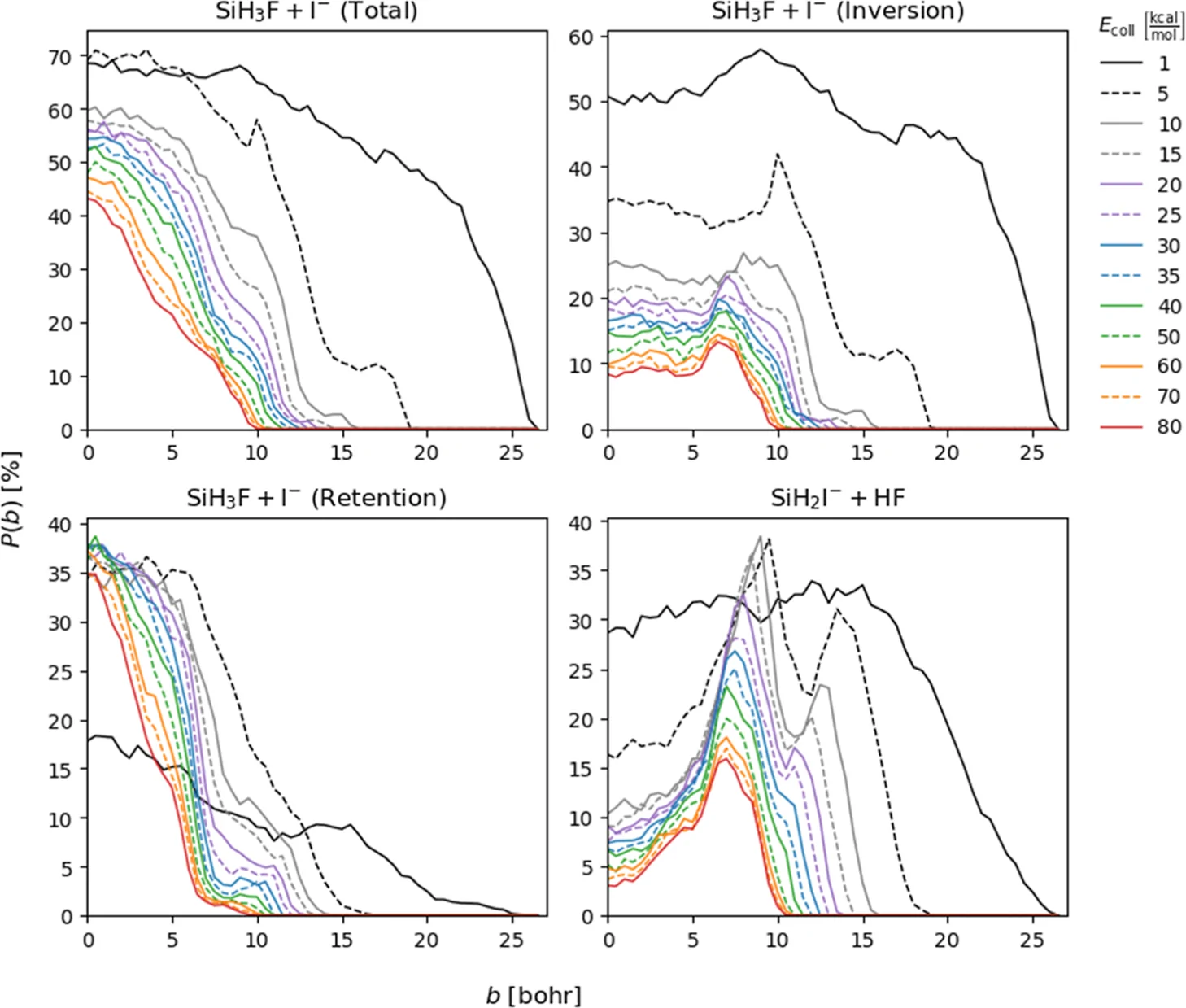
Reaction probabilities of the main channels of the SiH_3_I + F^–^ system as a function of impact parameter obtained at different collision energies between *E*_coll_ = 1 and 80 kcal/mol.

**Figure 4:**
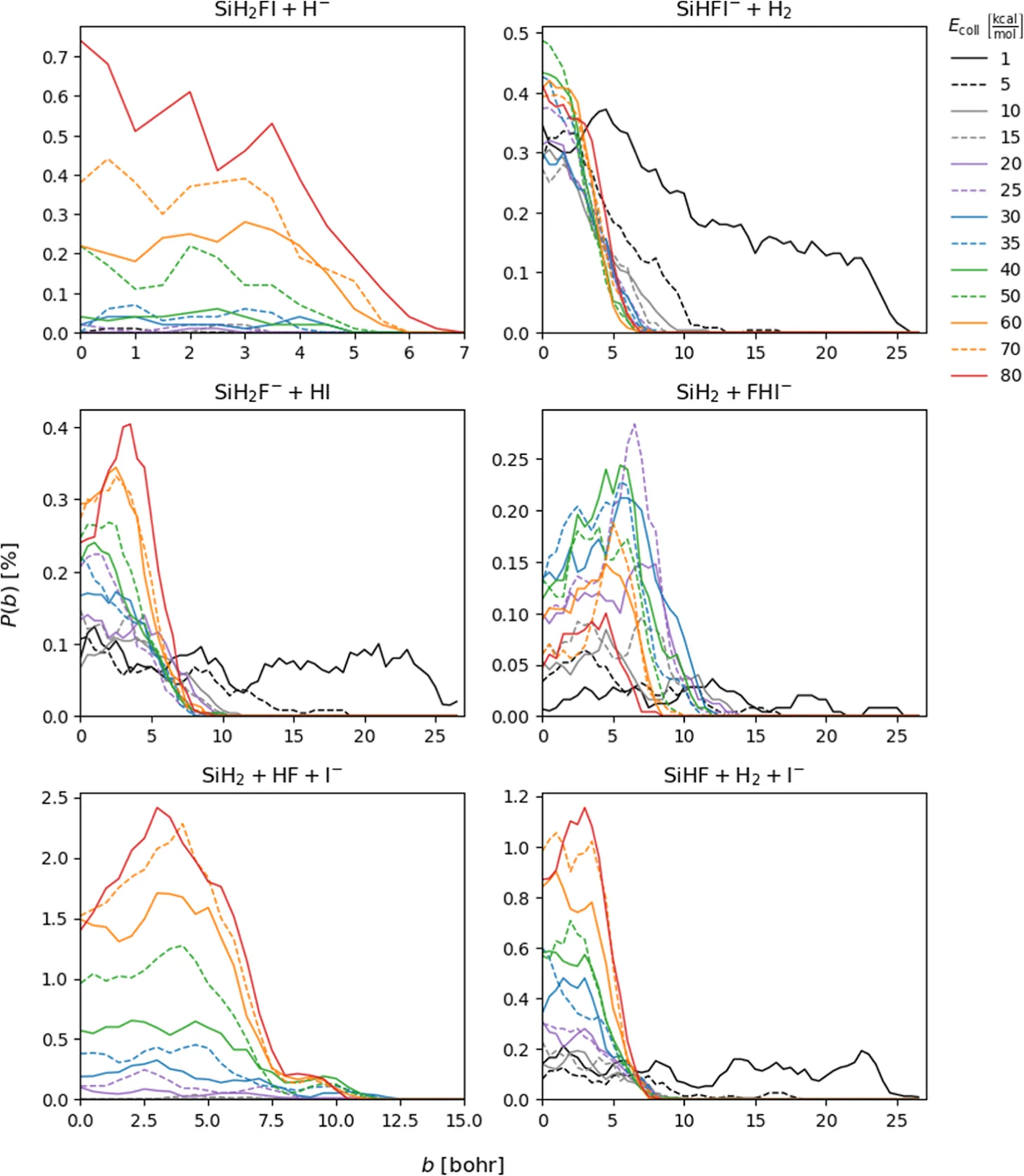
Reaction probabilities of the minor channels of the SiH_3_I + F^–^ system as a function of impact parameter obtained at different collision energies between *E*_coll_ = 1 and 80 kcal/mol. Moving averages are used to represent the curves.

**Figure 5:**
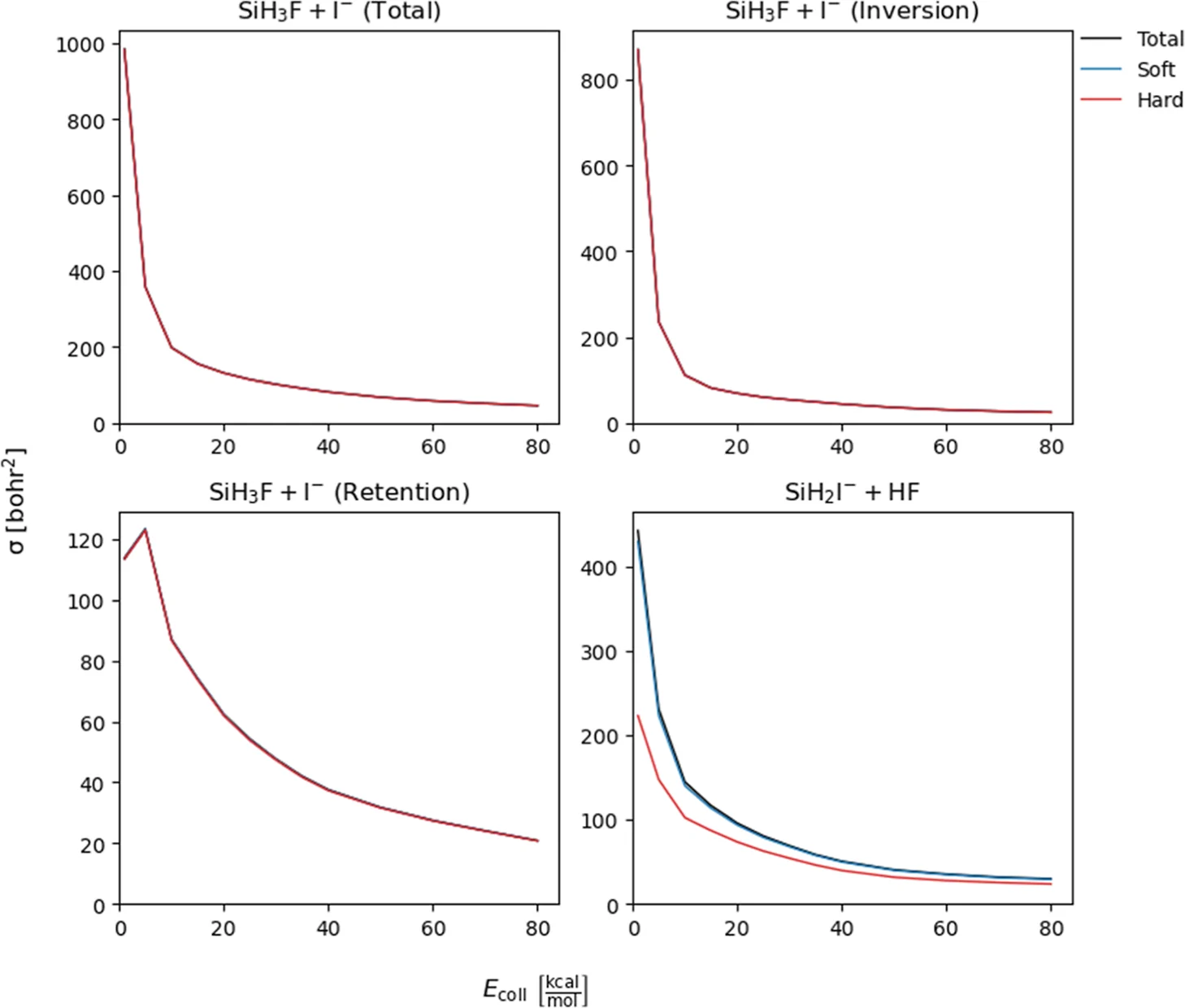
Integral cross sections of the main channels of the SiH_3_I + F^–^ system obtained at different collision energies between *E*_coll_ = 1 and 80 kcal/mol, with hard and soft vibrational energy constraints, as well as without energy constraint. Shaded regions indicate ±1 standard error, which is too small to be visually resolved at this scale.

**Figure 6:**
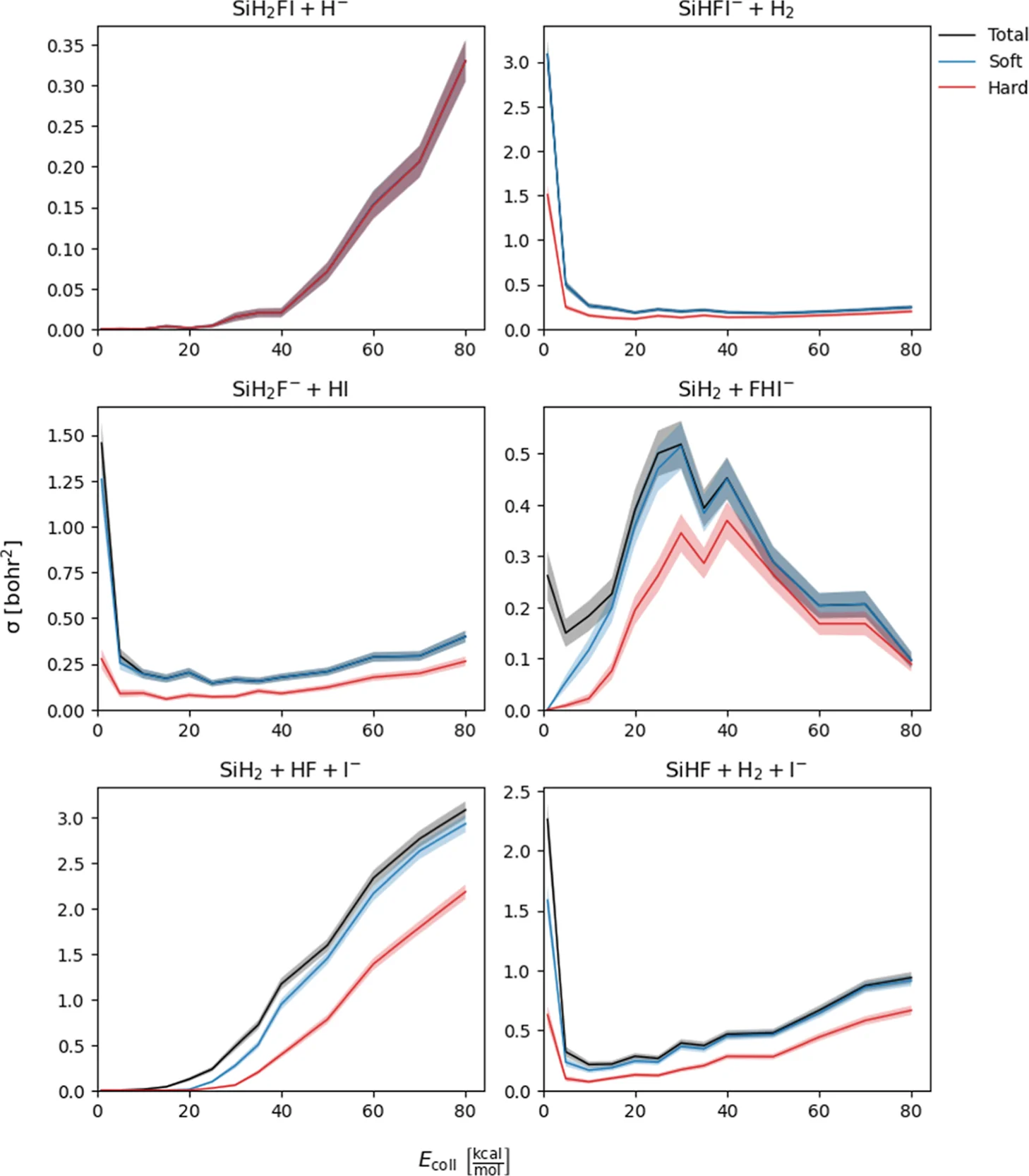
Integral cross sections of the minor channels of the SiH_3_I + F^–^ system obtained at different collision energies between *E*_coll_ = 1 and 80 kcal/mol, with hard and soft vibrational energy constraints, as well as without energy constraints. Shaded regions indicate ±1 standard error.

Across the investigated collision-energy range of *E*_coll_ = 1–80 kcal/mol the dominant reaction channel is iodide substitution, producing SiH_3_F + I^–^. Its ICS is extremely high in the low energy region, 990 bohr^2^ at *E*_coll_ = 1 kcal/mol, and decreases rapidly with increasing collision energy. Dividing the total SiH_3_F + I^–^ cross section into inversion and retention contributions shows strong inversion preference at low collision energy. At the lowest *E*_coll_, the inversion and retention ICSs are 870 and 110 bohr,^2^ respectively. The inversion–retention ratio drops sharply with the collision energy increase, from 7.7 at *E*_coll_ = 1 kcal/mol to 1.1–1.3 for collision energies between 10 and 80 kcal/mol. At the highest collision energy, the inversion and retention cross sections decrease to 25 and 21 bohr,^2^ respectively.

Vibrational constraints do not measurably affect the iodide substitution ICS. For inversion substitution, both the maximum reactive impact parameter and the reaction probability at *b* = 0 decrease monotonically with the collision energy increase as *b*_max_ falls from 26.5 to 10.5 bohr between *E*_coll_ = 1 and 80 kcal/mol, and *P*(*b* = 0) decreases from 51% to 8.4%, with the steepest change between *E*_coll_ = 1 and 5 kcal/mol. In the case of retention, at the lowest collision energy the probability decreases linearly from around 18% at *b* = 0 to zero at *b* = 26.5 bohr. For *E*_coll_ = 5–80 kcal/mol, *P*(*b* = 0) remains in a narrow range, between 34–38%, while the maximum reactive impact parameter decreases from 18.5 to 9.5 bohr. The large low-energy cross sections, particularly the high *b*_max_ values, indicate efficient capture by the long-range ion–dipole interaction. With increasing collision energy, the higher relative velocity reduces both the capture efficiency and the interaction time, narrowing the reactive impact-parameter range and consequently decreasing the ICSs.

The cross section profile and stereochemistry of iodide substitution closely resemble the analogous chloride substitution previously reported^29,31^ for SiH_3_Cl + F^–^. In the silyl chloride system, the total substitution ICS exceeds 1000 bohr^2^ at 1 kcal/mol, with contributions of approximately 900 and 120 bohr^2^ from inversion and retention, respectively, and the two contributions become comparable at high collision energies. By contrast, retention is a much less competitive process in CH_3_I + F^–^, where direct front-side substitution requires a positive-energy barrier and the lower-energy retention mechanism is double inversion.^10^

Proton abstraction (SiH_2_I^–^ + HF) is the second-most dominant reaction in the system and has an ICS of the same order of magnitude as iodide substitution. Its cross section decreases from around 440 bohr^2^ at *E*_coll_ = 1 kcal/mol to 30 bohr^2^ at 80 kcal/mol. The soft constraint has a negligible effect, whereas applying the hard constraint reduces the ICS obtained at *E*_coll_ = 1 kcal/mol by about a factor of 2. Fragment-resolved analysis shows that this reduction is mainly caused by the HF product, whose vibrational energy frequently falls below its ZPVE even when the total product vibrational energy satisfies the soft constraint (Figure S9). Violations of the SiH_2_I^–^ ZPVE are considerably less important. The maximum reaction probability of proton abstraction is 38% at an impact parameter of 9.5 bohr and a collision energy of 5 kcal/mol. The peak value decreases to 16% at the highest collision energy (80 kcal/mol), obtained at *b* = 7 bohr. At the lowest collision energy, *P*(*b*) remains in the 30–35% region over *b* = 0–16.5 bohr. Proton abstraction is somewhat more competitive in the silyl iodide system than in SiH_3_Cl + F^–^, for which the unconstrained low-energy ICS is approximately 350–370 bohr^2^.^31^ The contrast with CH_3_I + F^–^ is stronger, because proton abstraction is endothermic in the carbon-centered system but constitutes a major, barrierless channel for SiH_3_I + F^–^.^10^ Thus, both the silicon center and the iodine leaving group enhance this reaction.

All remaining channels have ICSs that are orders of magnitude lower. Without constraints, the SiHFI^–^ + H_2_ and SiH_2_F^–^ + HI productions reach σ = 3.1 and 1.5 bohr^2^ at 1 kcal/mol and fall below 0.5 bohr^2^ at collision energies above 10 kcal/mol. The SiH_2_ + FHI^–^ channel is favored at intermediate collision energy. Its cross section is peaking near 0.5 bohr^2^ at 30 kcal/mol. In contrast, SiH_2_ + HF + I^–^ and hydride substitution (SiH_2_FI + H^–^) are negligible at the lowest collision energy region but show increasing ICSs at high *E*_coll_, reaching σ = 3.1 and 0.33 bohr^2^ at *E*_coll_ = 80 kcal/mol. The SiHF + H_2_ + I^–^ production shows ICSs comparable to the cross sections of the SiHFI^–^ + H_2_ production throughout the entire collision energy range.

The broad maximum of the SiH_2_ + FHI^–^ cross section at intermediate collision energies is consistent with competing requirements of its multistep mechanism. At low collision energies, the halogen abstraction of HF is inefficient. At extremely high collision energies the shorter interaction time disfavors the formation of FBHMIN complexes and excess energy facilitates further dissociation of vibrationally excited FHI^–^ to HF and I^–^.

The effects of the vibrational constraints vary among the minor channels and generally become weaker with increasing collision energy. For SiH_2_ + HF + I^–^, the soft constraint halves the cross section at 30 kcal/mol but reproduces more than 90% of the unconstrained value above 50 kcal/mol. The reducing effect of the hard constraint remains stronger as the hard vibration constrained ICS is 50% of the unconstrained at 50 kcal/mol collision energy and around 71% at 80 kcal/mol. The probability of SiH_2_ + HF + I^–^ peaks near *b* = 3 bohr at high *E*_coll_, reaching 2.5% at 80 kcal/mol. For SiH_2_F^–^ + HI, the soft constraint has minor effect, while the hard constraint reduces the ICS to 20% of the unconstrained value at 1 kcal/mol, 50% at 40 kcal/mol, and 66% at 80 kcal/mol. For bihalide formation (SiH_2_ + FHI^–^), the unconstrained cross section is 0.26 bohr^2^ at 1 kcal/mol but drops to zero even under the soft constraint. The soft-constraint effect becomes negligible above *E*_coll_ = 20 kcal/mol, while the hard constraint halves the ICS near *E*_coll_ = 20 kcal/mol and decreases the unconstrained cross section by 10–20% above 40 kcal/mol. For the SiHF + H_2_ + I^–^ production channel the soft constrained ICS is around 70% of the total ICS at the lowest collision energy, and above 90% at collision energies exceeding 30 kcal/mol. The hard-constrained cross sections for this channel are significantly lower, yielding 28% and 70% of the unconstrained ICSs at *E*_coll_ = 1 and 80 kcal/mol, respectively.

Compared with SiH_3_Cl + F^–^, bihalide and three-fragment HF formation are substantially enhanced in the silyl iodide system. At 40 kcal/mol, the FHI^–^ channel reaches approximately 0.5 bohr^2^ compared with only 0.17 bohr^2^ for FHCl^–^, while the SiH_2_ + HF + Cl^–^ channel reaches merely 0.07 bohr^2^ and is strongly suppressed by the vibrational constraints. The corresponding SiH_2_ + HF + I^–^ channel has an ICS approximately 17 times as large as that of the chloride channel at *E*_coll_ = 40 kcal/mol.^31^

Overall, the excitation and opacity functions show that replacing Cl by I leaves the broad substitution dynamics of the silicon-centered reaction largely unchanged but increases the importance of proton abstraction, bihalide formation, and three-fragment HF production. Replacing C by Si has a more fundamental effect as retention substitution becomes a major reaction channel, while proton abstraction becomes a major barrierless process already at low collision energies, in contrast to CH_3_I + F^–^, where retention remains a minor pathway and proton abstraction is endothermic and becomes significant only at higher collision energies.^10^

### 3.3 Initial Attack Angle and Scattering Angle Distributions

Figures 7, 8, 9 and 10 show the distributions of the cosines of the initial reactant attack angles and the product scattering angles. In the inversion channel of the iodide-substitution reaction, at low collision energies between 1 and 5 kcal/mol, a clear back-side attack preference can be seen, characterized by a maximum at cos­(α) = –1. At higher collision energies, the cos­(α) distribution for inversion becomes approximately isotropic. In the retention channel, front-side attack preference is observed at *E*_coll_ = 1 kcal/mol. At higher collision energies, the preferred attacking direction shifts toward cos­(α) around −0.5, corresponding to an α angle of about 120°, and at the highest collision energies the front-side attacking direction, cos­(α) = 1, becomes the least favored. This behavior closely resembles that found previously for F^–^ + SiH_3_Cl, where inversion likewise changes from a pronounced low-energy back-side preference toward an approximately isotropic distribution, while the retention maximum shifts from the front side toward α ≈ 120° with increasing collision energy.^31^ The overall iodide-substitution channel, including both inversion and retention, is dominated by inversion at low *E*_coll_ because of its substantially larger ICS. As a result, the cos­(α) distribution shows a slight preference for back-side attack in the low-energy region. At higher collision energies, the distribution develops a maximum near cos­(α) ≈ –0.5, while both extremes, cos­(α) = –1 and 1, become less favorable. This evolution reflects both the changing channel-specific distributions and the increasing relative contribution of retention, whose ICS becomes comparable to that of inversion at high collision energies.

**Figure 7:**
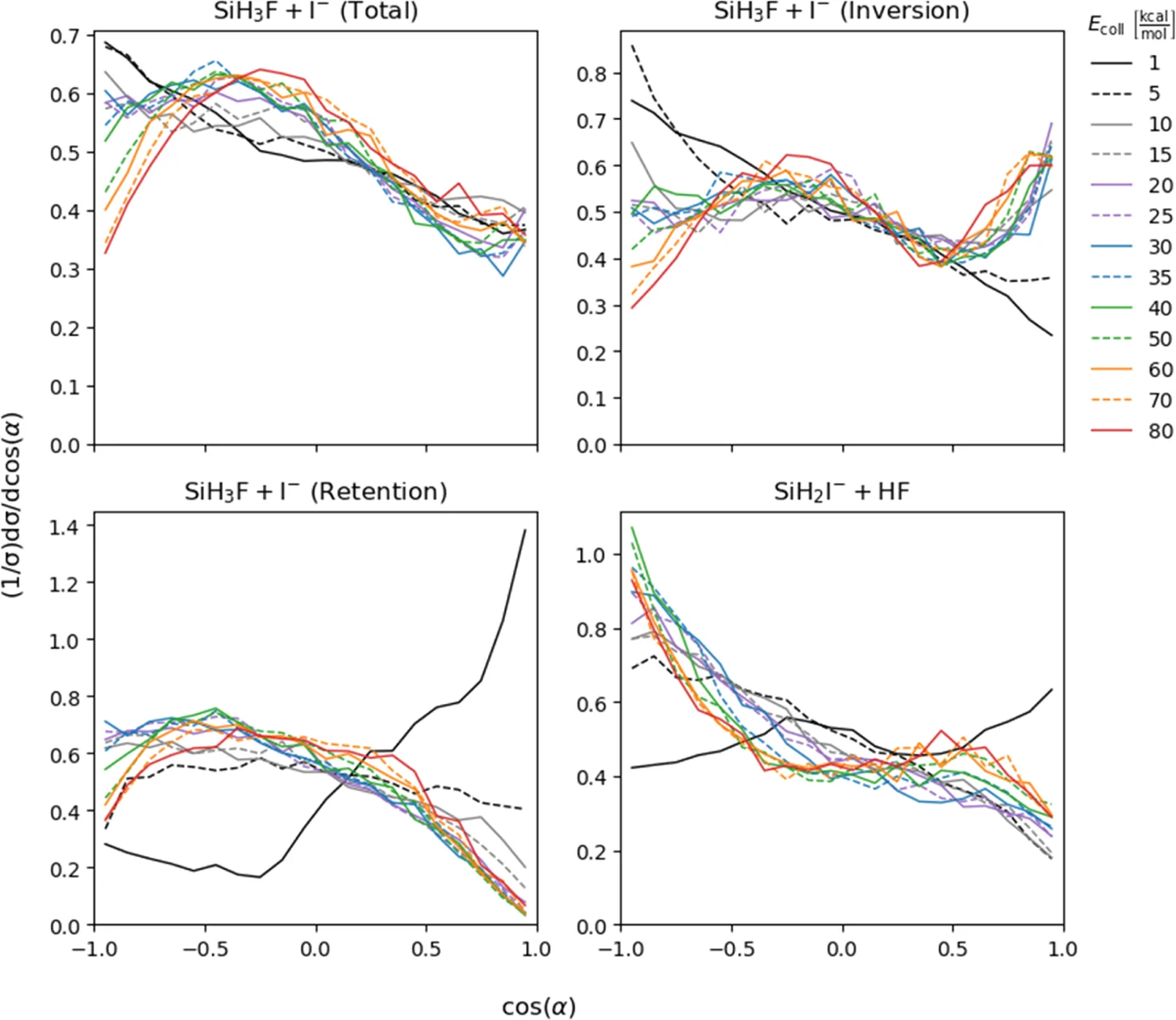
Initial attack angle distributions of the main channels of the SiH_3_I + F^–^ system obtained at different collision energies between *E*_coll_ = 1 and 80 kcal/mol.

**Figure 8:**
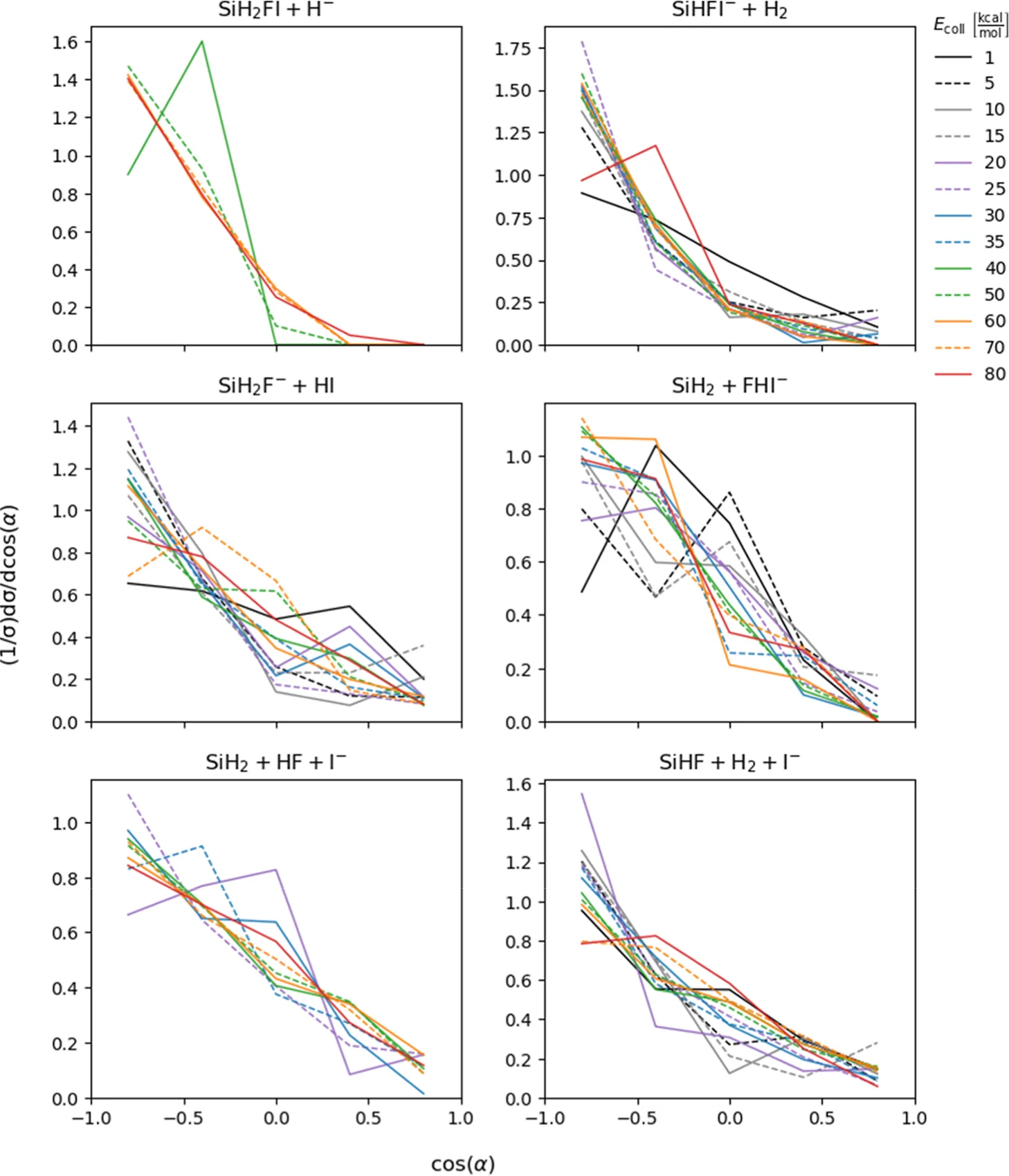
Initial attack angle distributions of the minor channels of the SiH_3_I + F^–^ system obtained at different collision energies between *E*_coll_ = 1 and 80 kcal/mol. Distributions are not shown for collision energies at which too few reactive trajectories were obtained for a statistically meaningful representation.

**Figure 9:**
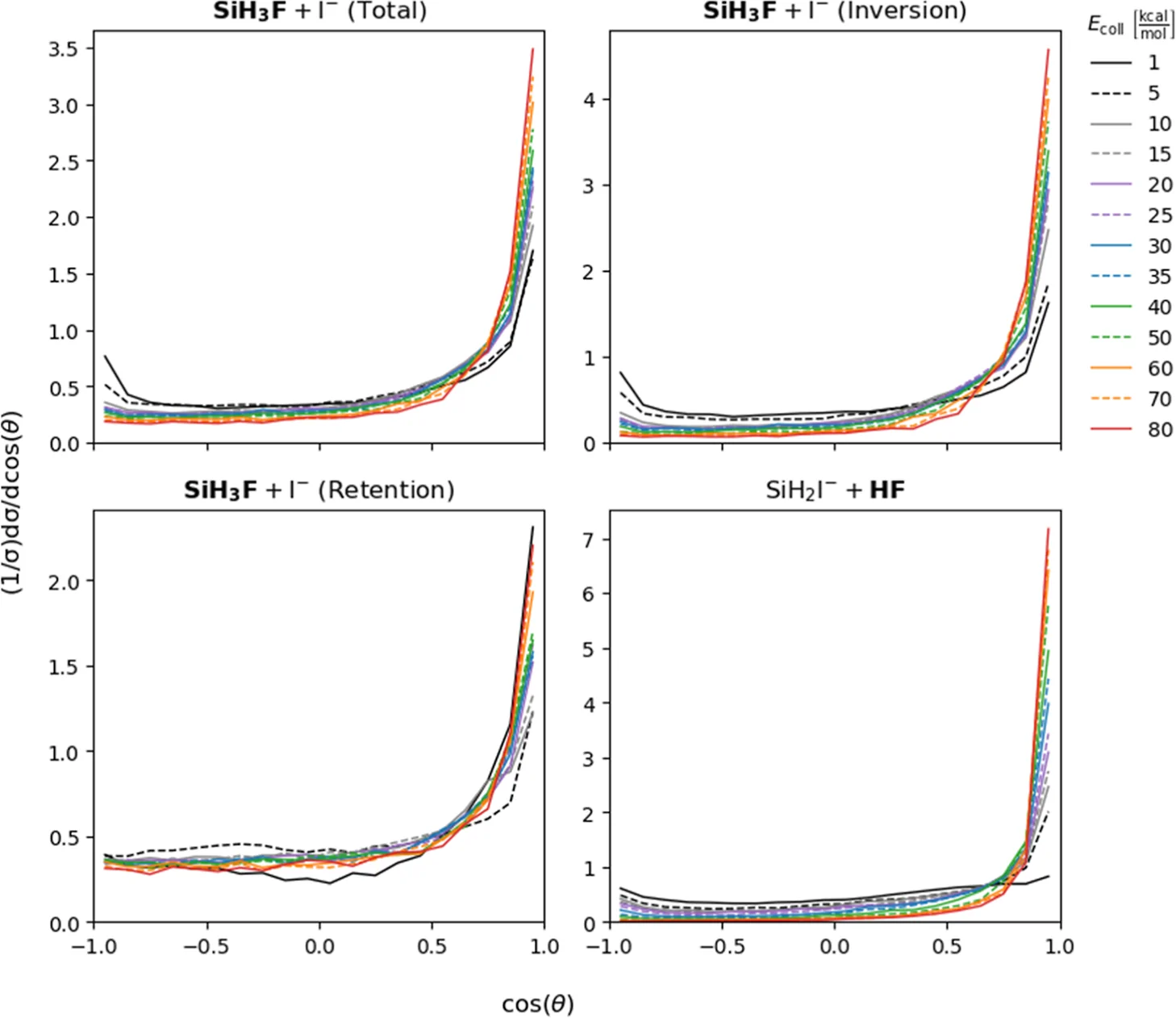
Scattering angle distributions of the main channels of the SiH_3_I + F^–^ system obtained at different collision energies between *E*_coll_ = 1 and 80 kcal/mol. Scattering angles are defined as the angle between the center-of-mass velocities of the F^–^ reactant and the product fragment in bold.

**Figure 10:**
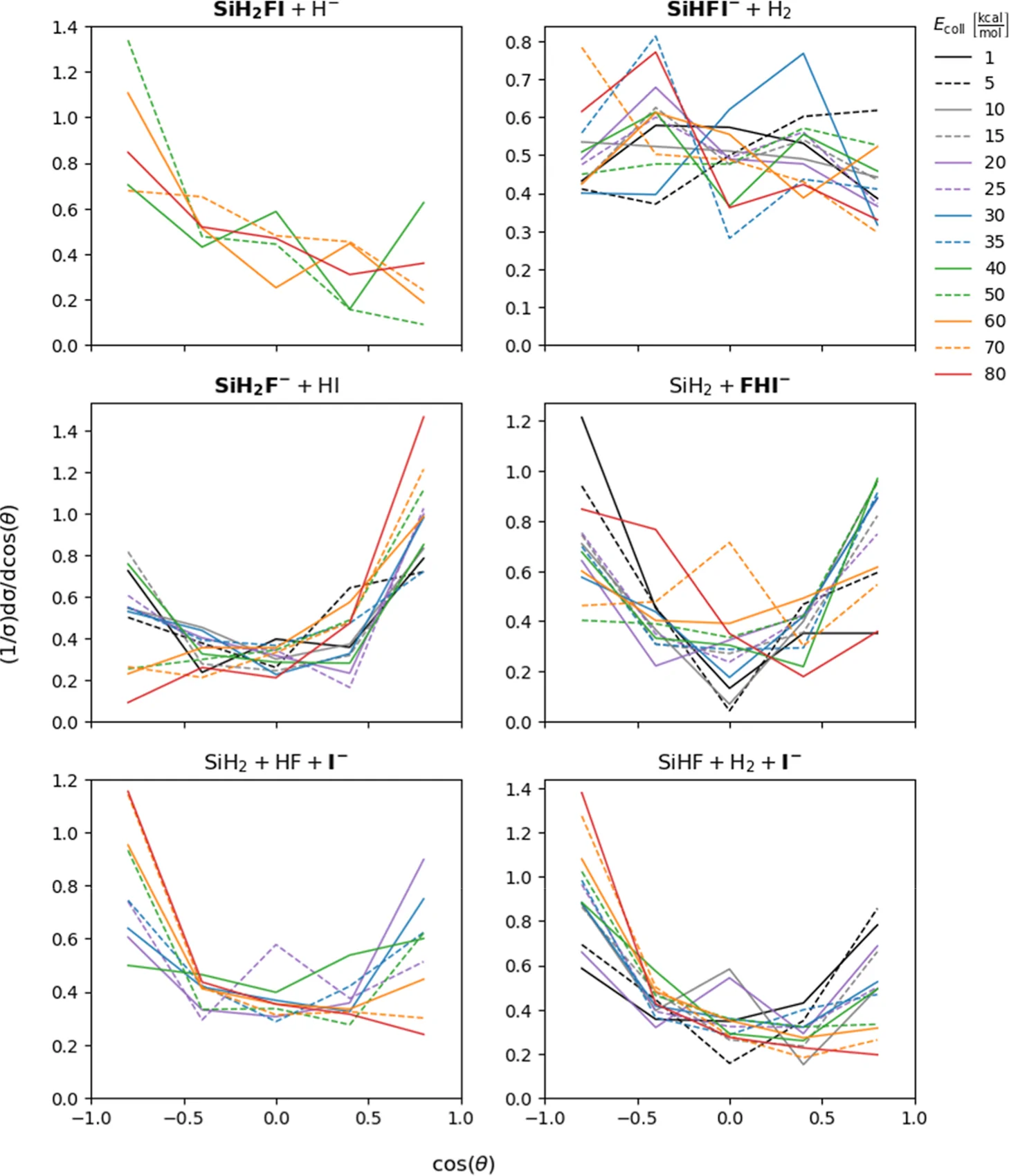
Scattering angle distributions of the minor channels of the SiH_3_I + F^–^ system obtained at different collision energies between *E*_coll_ = 1 and 80 kcal/mol. Scattering angles are defined as the angle between the center-of-mass velocities of the F^–^ reactant and the product fragment in bold. Distributions are not shown for collision energies at which too few reactive trajectories were obtained for a statistically meaningful representation.

At the lowest collision energy, substantial reorientation can occur during the long-range approach. In the analogous F^–^ + SiH_3_Cl system, we found that the initial attack angle and impact parameter jointly determine whether this motion mainly excites F–Si–Cl bending or overall rotation, thereby affecting the subsequent reaction pathway.^29,31^ Similar behavior occurs for F^–^ + SiH_3_I, where WMIN formation at 1 kcal/mol is common over a broad range of initial orientations. Consequently, the initial cos­(α) mainly characterizes the starting geometry rather than the short-range attack direction and contributes to the distinct low-energy attack-angle distributions. At higher collision energies, the shorter interaction time suppresses reorientation, making the initial attack angle more representative of the actual collision geometry.

The cos­(θ) scattering angle distributions for iodide substitution generally show a preference for forward scattering for both stereochemical outcomes, although the energy dependence differs. For inversion at low collision energy, forward scattering is only slightly favored, and the cos­(θ) distribution at *E*_coll_ = 1 kcal/mol is approximately symmetric. Forward scattering becomes more pronounced with increasing collision energy. In the retention substitution channel, forward scattering dominates at all collision energies, with the strongest forward scattering observed at 1 and 80 kcal/mol. Forward scattering for the retention pathway is least pronounced at *E*_coll_ = 5 kcal/mol.

Previous trajectory analysis of the title system showed that small-impact-parameter substitution can lead to backscattering, whereas larger-impact-parameter stripping trajectories preferentially scatter forward.^30^ The increasingly forward-scattered inversion products at higher energies are therefore consistent with a growing contribution from more direct reaction mechanisms. In contrast to the similar angular behavior of the two silicon-centered systems, carbon-centered F^–^ + CH_3_I shows substantially different retention dynamics. In the latter system, low-energy retention is dominated by the indirect double-inversion mechanism and direct front-side retention opens only at higher collision energies.^10^

For proton abstraction (SiH_2_I^–^ + HF), the initial attack angle distribution is nearly isotropic at the lowest collision energy and above *E*_coll_ = 5 kcal/mol back-side attack preference appears. The scattering angle distributions indicate a slight forward scattering preference at *E*_coll_ = 1 kcal/mol, which develops into an extremely strong forward-scattering preference at higher energies. Previous trajectory analysis showed that at *E*_coll_ = 1 kcal/mol and small impact parameters proton abstraction frequently proceeds after WMIN formation, whereas at larger impact parameters a more direct stripping mechanism without Si–F bond formation leads to forward scattering.^30^ The increasingly strong forward-scattering preference found here therefore provides ensemble-level support for the growing importance of the direct mechanism. The corresponding SiH_3_Cl reaction shows a closely related high-energy trend, changing toward back-side initial attack and forward scattering with increasing collision energy. However, at the lowest collision energy, proton abstraction in the silyl chloride system shows a clear front-side attack preference,^31^ whereas the distribution of the title system is close to being isotropic.

For the SiH_2_ + HF + I^–^ production channel, the cos­(α) distributions between *E*_coll_ = 20 and 80 kcal/mol consistently show a back-side attack preference. Because of low reaction probability, distributions at lower collision energies could not be obtained. The cos­(θ) distributions reveal a backscattering preference at high collision energies, while at lower energies, near *E*_coll_ = 20 kcal/mol, the scattering becomes more symmetric and nearly isotropic. Because this three-fragment channel can arise through several multistep pathways including FHI^–^ decomposition, iodide elimination following proton abstraction, and HF elimination following iodide substitution,^30^ its final scattering distribution reflects the dynamics of the subsequent fragmentation steps.

For the remaining channels, SiH_2_FI + H^–^, SiHFI^–^ + H_2_, SiH_2_F^–^ + HI, SiH_2_ + FHI^–^, and SiHF + H_2_ + I^–^ the cos­(α) distributions are similar and consistently show a back-side attack preference. For the hydride-substitution channel, SiH_2_FI + H^–^, data below 50 kcal/mol were not plotted because of low reaction probability. Similarly, for the SiH_2_ + FHI^–^ production, distributions at the lowest collision energy of 1 kcal/mol could not be shown. For multistep reactions, the initial attack-angle distributions primarily reflect the angle preferences of the precursors. For example, SiH_2_F^–^ + HI is formed predominantly by iodide substitution followed by proton abstraction of the leaving ion, with frequent postreaction IBHMIN formation that can modify the fragment departure direction. SiH_2_ + FHI^–^, in contrast, proceeds through proton abstraction followed by FBHMIN formation and decomposition into SiH_2_ + FHI^–^.^30^ Thus, similar initial attack-angle distributions do not necessarily imply similar subsequent dynamics.

The hydride-substitution reaction favors backward scattering. The SiHFI^–^ + H_2_ channel exhibits isotropic scattering without a preferred direction. The SiH_2_F^–^ + HI channel shows symmetric forward and backward scattering at low collision energies, which changes into forward scattering preference as the collision energy increases.

In the SiH_2_ + FHI^–^ channel, the scattering distribution is symmetric at low collision energy and develops a backscattering preference at *E*_coll_ = 80 kcal/mol. This is notable because its precursor, proton abstraction, becomes strongly forward scattered at high collision energies. The change in scattering direction suggests that FBHMIN formation and decomposition can partly erase or even reverse the directional preference of the preceding proton-abstraction step. A similar contrast between forward-scattered proton abstraction and backward-scattered bihalide formation was previously observed for the analogous F^–^ + SiH_3_Cl system.^31^

The H_2_-producing channels involve several mechanisms whose relative importance changes with collision energy. SiHFI^–^ + H_2_ can form through IDHBMIN decomposition and reassociation at low energy, H_2_ elimination from WMIN, FSPostMIN, or vibrationally excited SiH_3_F, and proton abstraction by H^–^ at higher energy ranges. The dominant mechanism of SiHF + H_2_ + I^–^ also changes with collision energy.^30^ Therefore, their angular distributions reflect contributions from several pathways rather than a single reaction mechanism.

Overall, the angular distributions show that the observed directionality is determined by both the initial collision geometry and subsequent complex-mediated dynamics. Their mechanistic interpretation is therefore most informative when combined with the trajectory analysis and the product-energy distributions discussed below.

### 3.4 Product Energy Distributions

Figures 11 and 12 show the translational energy distributions of the SiH_3_I + F^–^ reactions in the collision-energy range between 1 and 80 kcal/mol. These distributions provide information about the directness of the individual reaction channels. Because product translation and internal excitation share the fixed available energy, their distributions provide complementary views of the same energy partitioning. Therefore, only the translational energy distributions are shown in the main text, while the internal, vibrational, and rotational distributions are provided in the Supporting Information (Figures S3–S8).

**Figure 11:**
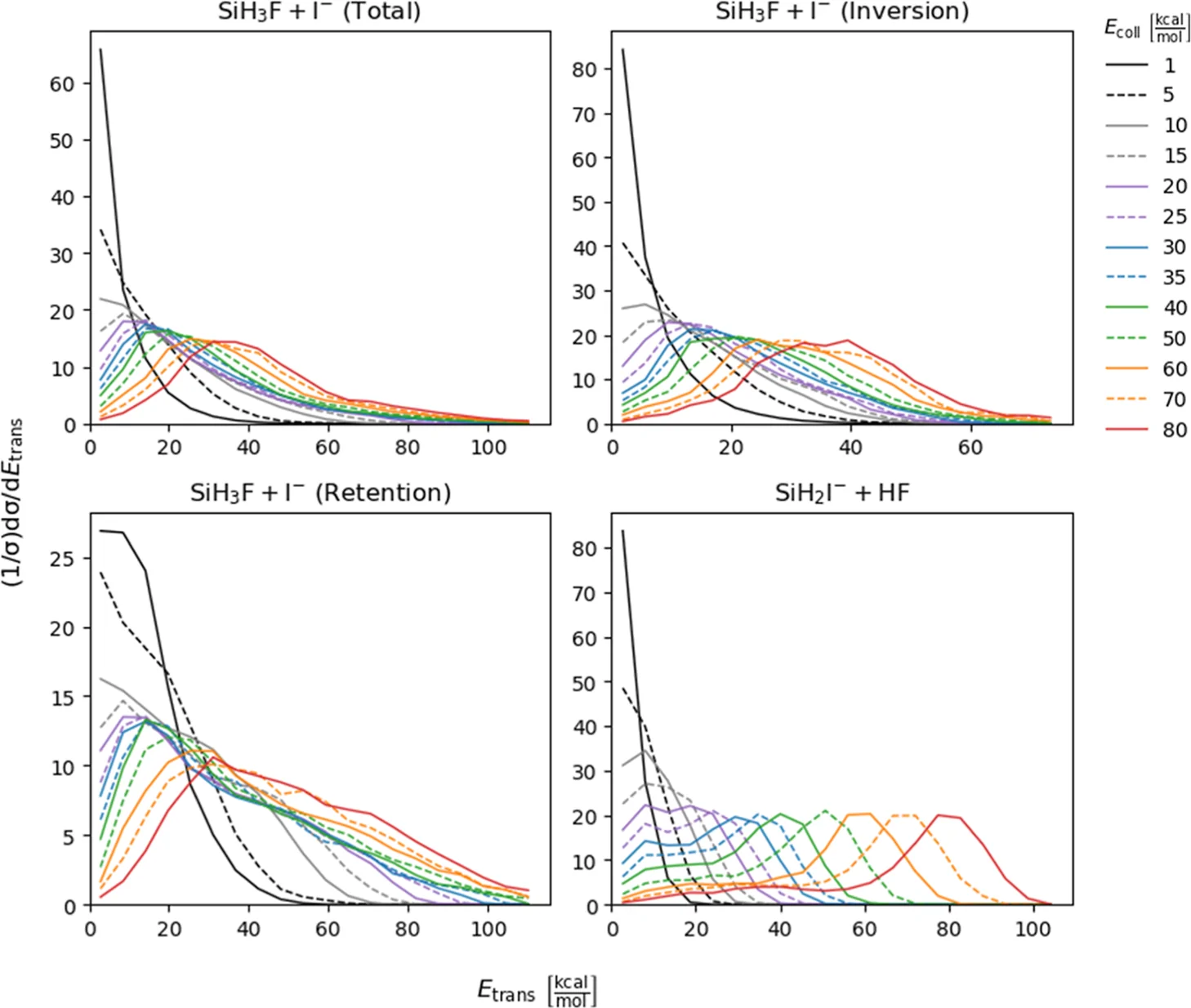
Translational energy distributions of the main channels of the SiH_3_I + F^–^ system obtained at different collision energies between *E*_coll_ = 1 and 80 kcal/mol.

**Figure 12:**
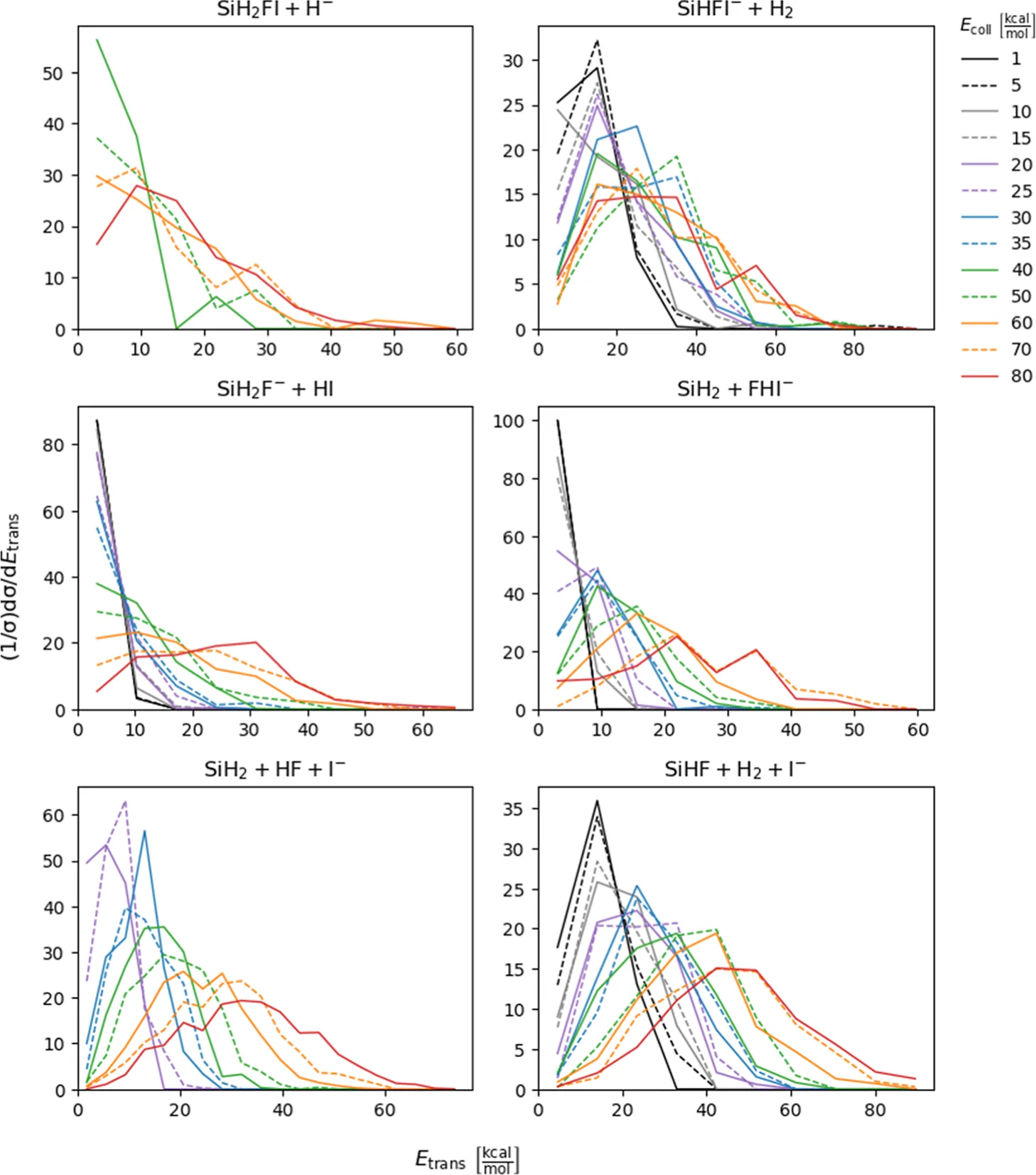
Translational energy distributions of the minor channels of the SiH_3_I + F^–^ system obtained at different collision energies between *E*_coll_ = 1 and 80 kcal/mol. Distributions are not shown for collision energies at which too few reactive trajectories were obtained for a statistically meaningful representation.

The distributions are normalized separately for each product channel and collision energy. Their peak heights characterize the shape of the energy distribution rather than the relative importance of the corresponding reaction channels, which is quantified by the ICSs. For the lowest-probability channels, fine structure in the normalized distributions should be interpreted with caution because of the smaller number of reactive trajectories.

Throughout the simulations, total energy is conserved to numerical accuracy and can be expressed as the sum of the reactant zero-point vibrational energy and the collision energy, reduced by the reaction energy defined by the relative energies of the products on the potential energy surface. For example, for the iodide substitution, the available energy at *E*_coll_ = 1 and 80 kcal/mol is 88 and 167 kcal/mol, respectively. For a given product channel, the increase in available energy with *E*_coll_ is therefore partitioned between product translation and internal excitation. The distributions show how this partitioning changes with collision energy.

The energy distributions of iodide substitution indicate mixed direct and indirect character, as the maxima of the translational energy distributions shift by roughly half of the increase in collision energy for both inversion and retention channels. The internal-energy maxima undergo a shift of comparable magnitude, showing that both product translation and internal excitation increase substantially with *E*_coll_ rather than either component remaining nearly unchanged.

The vibrational energy distribution peaks of the substitution channel change much less than the total internal energy and remain broadly in the same energy region, with a moderate shift and substantial broadening as *E*_coll_ increases. In contrast, the rotational distributions shift systematically toward higher energies with increasing *E*_coll_, particularly for inversion. The much stronger shift of the rotational distributions shows that increasing rotational excitation contributes importantly to the collision-energy dependence of *E*_int_.

At low collision energies, the large ICSs and the extremely high *b*_max_ indicate a capture-driven process governed by long-range interactions. In contrast, at higher collision energies, the decreasing cross sections and maximum reactive impact parameters, as well as increasingly forward-scattering products, suggest the emergence of more direct mechanisms. Higher collision energies are expected to shorten the interaction time and reduce the opportunity for complex formation, consistent with the increasing direct contribution. The inversion and retention cross sections also become more similar at high *E*_coll_.

Comparison with the analogous SiH_3_Cl + F^–^ and CH_3_I + F^–^ reactions shows clear differences in substitution dynamics. For SiH_3_Cl + F^–^, the translational-energy maximum remains below 10 kcal/mol throughout the investigated 1–40 kcal/mol range, while most of the available energy is deposited into internal excitation, consistent with predominantly indirect substitution.^31^ In CH_3_I + F^–^, indirect mechanisms likewise dominate at low collision energies, where the internal-energy distributions are concentrated near the upper end of the energetically accessible range. At higher collision energies, however, direct rebound and stripping become increasingly important and eventually dominate this carbon-centered substitution.^10^ The present SiH_3_I + F^–^ system also shows an increasing direct contribution with collision energy, but its energy partitioning differs from that of SiH_3_Cl + F^–^, as the translational-energy distributions shift much more strongly with *E*_coll_. Although the substitution ICSs and stereochemical trends of the two silicon-centered systems are similar, their product-energy partitioning reveals a leaving-group effect.

For the proton-abstraction reaction (SiH_2_I^–^ + HF), the maxima of the translational energy distributions shift toward higher energies as the collision energy increases. However, an additional, less pronounced bump remains at lower translational energies and does not follow the *E*_coll_ increase. This pattern is consistent with coexisting direct and indirect contributions, with the direct component becoming increasingly dominant as *E*_coll_ increases. This interpretation is consistent with the angular distributions, which show increasingly strong forward scattering and were associated with a growing contribution from direct stripping trajectories at high collision energy. The internal energy distributions of the proton-abstraction channel support this interpretation. The main maximum remains in the 30–40 kcal/mol range across the investigated collision energies, which is close to the total available energy (35 kcal/mol) for proton abstraction excluding the collision energy. At higher collision energies, a secondary feature appears at elevated internal energies corresponding to the smaller contribution of the indirect mechanism. The vibrational and rotational energy distributions provide further details. The vibrational energy maxima remain near 30 kcal/mol, while the rotational maxima stay below 5 kcal/mol across the entire collision energy range. At low collision energies, most of the available energy appears as product vibrational excitation. Because neither the vibrational nor rotational maximum follows the increase in *E*_coll_, most of the additional collision energy appears in product translation. This provides a clearer signature of direct mechanism than in iodide substitution. A similar two-component translational-energy distribution was reported for proton abstraction in SiH_3_Cl + F^–^, suggesting that replacement of Cl by I does not qualitatively change the basic energy-partitioning behavior of this channel.^31^ At *E*_coll_ = 1 kcal/mol, both the soft and hard vibrational constraints have only minor effects on the shapes of the normalized angular and product-energy distributions (Figure S10), despite the substantial reduction of the absolute cross section by the hard constraint. Thus, the mechanistic conclusions drawn from the unconstrained distributions remain essentially unchanged.

Hydride ion substitution involves complex formation, sharing the WMIN and FSPostMIN minima with the main substitution pathway, while other low-probability reactions (SiH_2_ + HF + I^–^, SiHF + H_2_ + I^–^, SiHFI^–^ + H_2_, SiH_2_F^–^ + HI and SiH_2_ + FHI^–^) proceed by multistep mechanisms. Such pathways provide greater opportunity for energy redistribution, although the relative partitioning between translation and internal excitation depends strongly on the specific mechanism and collision energy.

For SiH_2_ + HF + I^–^ and SiHF + H_2_ + I^–^ formation channels, both translational and internal excitation increase systematically with collision energy, but the detailed partitioning differs between the two channels. For SiH_2_ + HF + I^–^, substantial shifts occur in both *E*_trans_ and *E*_int_, indicating that the additional collision energy is shared between fragment separation and internal excitation. Its rotational distribution remains concentrated at comparatively low energies, whereas the vibrational distribution shifts much more strongly, showing that most of the increase in internal energy is vibrational. SiHF + H_2_ + I^–^ shows a similar overall increase in both translational and internal energies, but with broad distributions consistent with contributions from several multistep mechanisms identified in the trajectory analysis.^30^ Similarly, the increase in internal energy is dominated by vibrational rather than rotational excitation.

In the case of hydride substitution (SiH_2_FI + H^–^), reliable statistics are available only above *E*_coll_ = 35 kcal/mol. Between 40 and 80 kcal/mol, the internal energy maximum increases consistently with the collision energy, whereas the translational energy maximum remains below 10 kcal/mol even at the highest collision energy, consistent with indirect reactions. The vibrational-energy distributions account for a substantial part of this increase in internal excitation, whereas the rotational distributions remain comparatively low and are less systematic. Thus, hydride substitution represents a contrast to the translation-dominated proton-abstraction channel. This behavior closely resembles hydride substitution in SiH_3_Cl + F^–^, where the available energy is mostly stored internally, predominantly as vibration.^31^ The strongly indirect energy-partitioning of this channel therefore appears to be largely preserved upon replacement of Cl by I.

For the SiHFI^–^ + H_2_ formation, the internal energy maximum shifts from approximately 55 kcal/mol at the lowest collision energies to roughly 110–120 kcal/mol at the highest energies, corresponding to an increase of about 60 kcal/mol. The translational energy distribution maximum shifts more moderately, remaining mainly in the approximately 15–30 kcal/mol region. Thus, most of the increase in available energy is accommodated by internal excitation rather than by a comparable increase in product translation. The vibrational distributions show a particularly strong shift with collision energy, whereas rotational excitation remains a smaller contribution. The substantial product translation already present at low collision energy originates from the exothermicity of the overall product-forming process and should not be interpreted as conversion of collision energy alone.

For the SiH_2_F^–^ + HI channel the translational energy maximum remains below 10 kcal/mol at collision energies lower than 60 kcal/mol, consistent with indirect character. However, between *E*_coll_ = 60 and 80 kcal/mol, an approximately 20 kcal/mol shift occurs, suggesting a mechanism change at extremely high collision energies, which allows internal energy transfer to product translation. The distributions are narrow at low and intermediate collision energy. At *E*_coll_ = 1 kcal/mol, nearly all internal energies lie between 10 and 30 kcal/mol, at *E*_coll_ = 20 kcal/mol, between 30 and 45 kcal/mol, and at *E*_coll_ = 80 kcal/mol most of the trajectories exceed 40 kcal/mol internal energy. The strong systematic shift of the vibrational distributions shows that vibrational excitation accounts for much of this increase in *E*_int_, while the rotational contribution remains substantially smaller.

For SiH_2_ + FHI^–^ formation, the translational energy maximum shifts to about 20 kcal/mol over the 80 kcal/mol collision-energy interval. At low collision energies, both the translational and internal energy distributions are extremely narrow. At *E*_coll_ = 1 kcal/mol, almost the entire internal energy distribution of SiH_2_ + FHI^–^ formation falls between 10 and 20 kcal/mol, for *E*_coll_ = 20 kcal/mol, between 20 and 40 kcal/mol, and the distributions gradually broaden at higher collision energies. The simultaneous increase in translational and internal energies shows that the additional collision energy is shared between product separation and internal excitation. The increase in internal energy is dominated mainly by vibration, while rotational excitation remains smaller. A qualitatively similar collision-energy-dependent shift of the translational distribution was reported for the analogous SiH_2_ + FHCl^–^ channel.^31^ Thus, replacing Cl by I makes bihalide formation thermochemically more favorable and substantially increases its cross section, while the two systems retain qualitatively similar energy-partitioning trends, with increasing collision energy appearing in both product translation and internal excitation.

The product-energy distributions show that the different channels partition the available energy in different ways. Proton abstraction is the most strongly translation-dominated major channel: its internal-energy distribution changes only modestly with collision energy, while most of the additional energy appears in product translation, consistent with the increasing importance of forward-scattered direct stripping. Iodide substitution shows a more balanced partitioning, with the increase in collision energy shared between translation and internal excitation, and the latter increasingly includes rotational excitation. Hydride substitution and several multistep minor channels show the opposite tendency, retaining a larger fraction of the additional energy internally, mainly as vibration. Taken together with the opacity, angular, and trajectory analyses, these distributions provide an independent picture of how the available energy is divided among the product degrees of freedom and how this partitioning changes across the different reaction pathways.

## 4 Summary and Conclusions

We have presented a detailed analysis of the quasi-classical trajectory ensemble previously generated for the F^–^ + SiH_3_I reaction on a full-dimensional analytical potential energy surface. Reaction probabilities and integral cross sections were combined with initial attack-angle, scattering-angle, and product-energy distributions over the *E*_coll_ = 1–80 kcal/mol range. This analysis provides a unified description of the stereochemistry, directionality, and energy partitioning of the major and minor reaction channels and allows the present results to be compared with previous studies of the analogous SiH_3_Cl + F^–^ and CH_3_I + F^–^ systems.

Iodide substitution is the dominant reaction throughout the investigated energy range. Its cross section is exceptionally large at low collision energy and decreases rapidly with increasing *E*_coll_. Walden inversion strongly dominates at *E*_coll_ = 1 kcal/mol, but the inversion and retention cross sections become comparable at higher energies. The large low-energy reactive impact parameters and frequent WMIN formation are consistent with capture-dominated, complex-mediated substitution. With increasing collision energy, the reactive impact-parameter range decreases and the products become increasingly forward scattered, consistent with a reduced contribution from complex-mediated pathways. The attack-angle distributions also change substantially with collision energy: the pronounced low-energy back-side preference of inversion is largely lost, while the preferred initial orientation for retention shifts away from the front side. Product-energy distributions reveal an additional change that is not evident from the cross sections alone. Both translational and internal excitation increase substantially with *E*_coll_, with the rotational contribution becoming increasingly important, particularly for inversion.

Proton abstraction is the second major reaction channel and shows a different evolution with collision energy. At low collision energy, its broad impact-parameter dependence and previously identified WMIN-mediated trajectories indicate a substantial contribution from complex-mediated pathways.^30^ At higher *E*_coll_, the scattering becomes strongly forward directed and the translational-energy distribution shifts markedly toward higher energies, consistent with an increasing contribution from stripping. In contrast, the main internal-, vibrational-, and rotational-energy features change much less with collision energy. Thus, among the major channels, proton abstraction shows the clearest increase in product translation as *E*_coll_ increases.

The lower-probability channels display a wider range of behavior. Hydride substitution remains strongly associated with internal excitation and provides the clearest contrast to proton abstraction. The H_2_, HI, bihalide, and three-fragment product formation channels proceed through several multistep pathways, and their angular and energy distributions consequently reflect combinations of precursor reactions and subsequent rearrangement or fragmentation. In particular, bihalide formation shows a pronounced change in scattering direction relative to its proton-abstraction precursor, indicating that intermediate formation and decomposition can substantially modify the directional information inherited from an earlier reaction step. Product-energy distributions further show that several of these channels accommodate a substantial part of the increasing available energy through vibrational excitation rather than translation alone.

Comparison with previous studies of related systems helps place the present results in the context of leaving-group and central-atom effects. Replacing Cl by I leaves the broad substitution cross-section and stereochemical trends of the silicon-centered reaction largely unchanged, but proton abstraction becomes more important and bihalide and three-fragment HF formation are substantially enhanced. The product-energy distributions nevertheless reveal a clear difference in substitution energy partitioning, because the translational distributions of SiH_3_I + F^–^ shift much more strongly with collision energy than those reported for SiH_3_Cl + F^–^.^31^ In contrast to CH_3_I + F^–^, the deeply submerged silicon-centered substitution pathways make both inversion and retention accessible throughout the investigated energy range, while proton abstraction is a major barrierless reaction rather than an endothermic channel that becomes significant only at higher collision energies.^10^

Taken together, the results show that the F^–^ + SiH_3_I reaction cannot be described by a single mechanistic picture. Collision energy changes the relative importance of capture, complex formation, stripping, rearrangement, and fragmentation differently for each product channel. The combination of cross sections, opacity functions, angular distributions, trajectory analysis, and product-energy partitioning therefore provides a more complete picture of the channel-specific dynamics of this multichannel ion–molecule reaction than any of these observables alone.

## Supplementary Material



## Notes

Reaction probabilities with standard errors as well as internal energy, vibrational energy, and rotational energy distributions for the major and minor channels of the F^–^ + SiH_3_I reaction at different collision energies. At the lowest collision energy correlated product vibrational-energy distribution as well as angular and energy distributions without and with ZPVE constraints are also given for the proton-abstraction channel (PDF)
