# Supplementary material for: Detailed Quasi-Classical Dynamics of the F– + SiH3I Multi-Channel Reaction
Source: J Phys Chem A. 2026 Oct 23;130(44):8858–74. doi: 10.1021/acs.jpca.6c05884 (PMC13746812; doi:10.1021/acs.jpca.6c05884)
Supplement: Supplementary file 1 [file jp6c05884_si_001.pdf]

SUPPORTING INFORMATION

Detailed Quasi-Classical Dynamics of the F^- + SiH_3I Multi-Channel Reaction

Attila Á. Dékány, Balázs J. Molnár, and Gábor Czakó*

MTA-SZTE Lendület "Momentum" Computational Reaction Dynamics Research Group, Interdisciplinary Excellence Centre and Department of Physical Chemistry and Materials Science, Institute of Chemistry, University of Szeged, Rerrich Béla tér 1, Szeged H-6720, Hungary

* E-mail: gczako@chem.u-szeged.hu

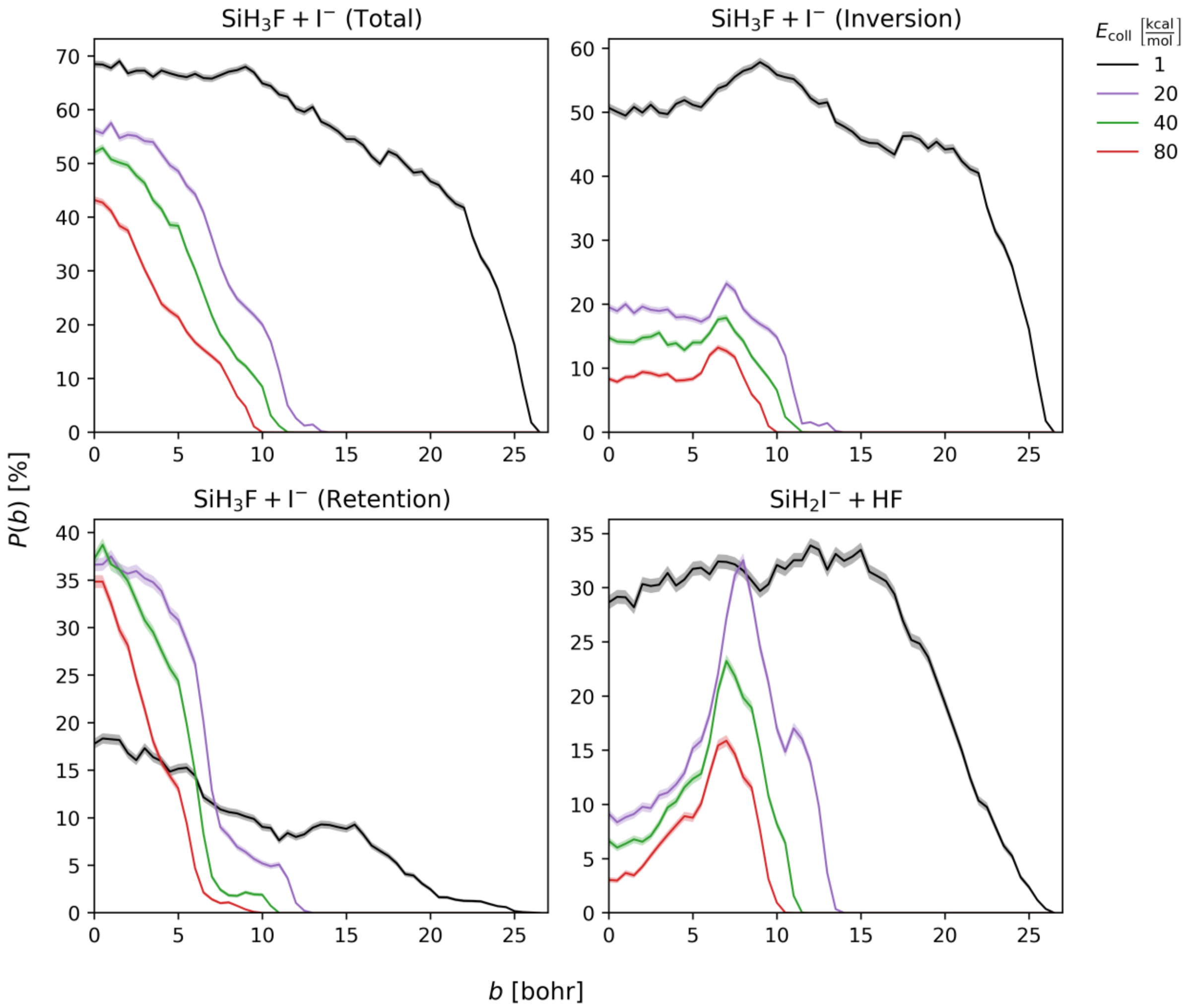

Figure S1. Reaction probabilities of the major channels of the $SiH_3I + F^-$ system as a function of impact parameter obtained at selected collision energies between E_{coll} = 1 and 80 kcal/mol. Shaded regions indicate ±1 standard error.

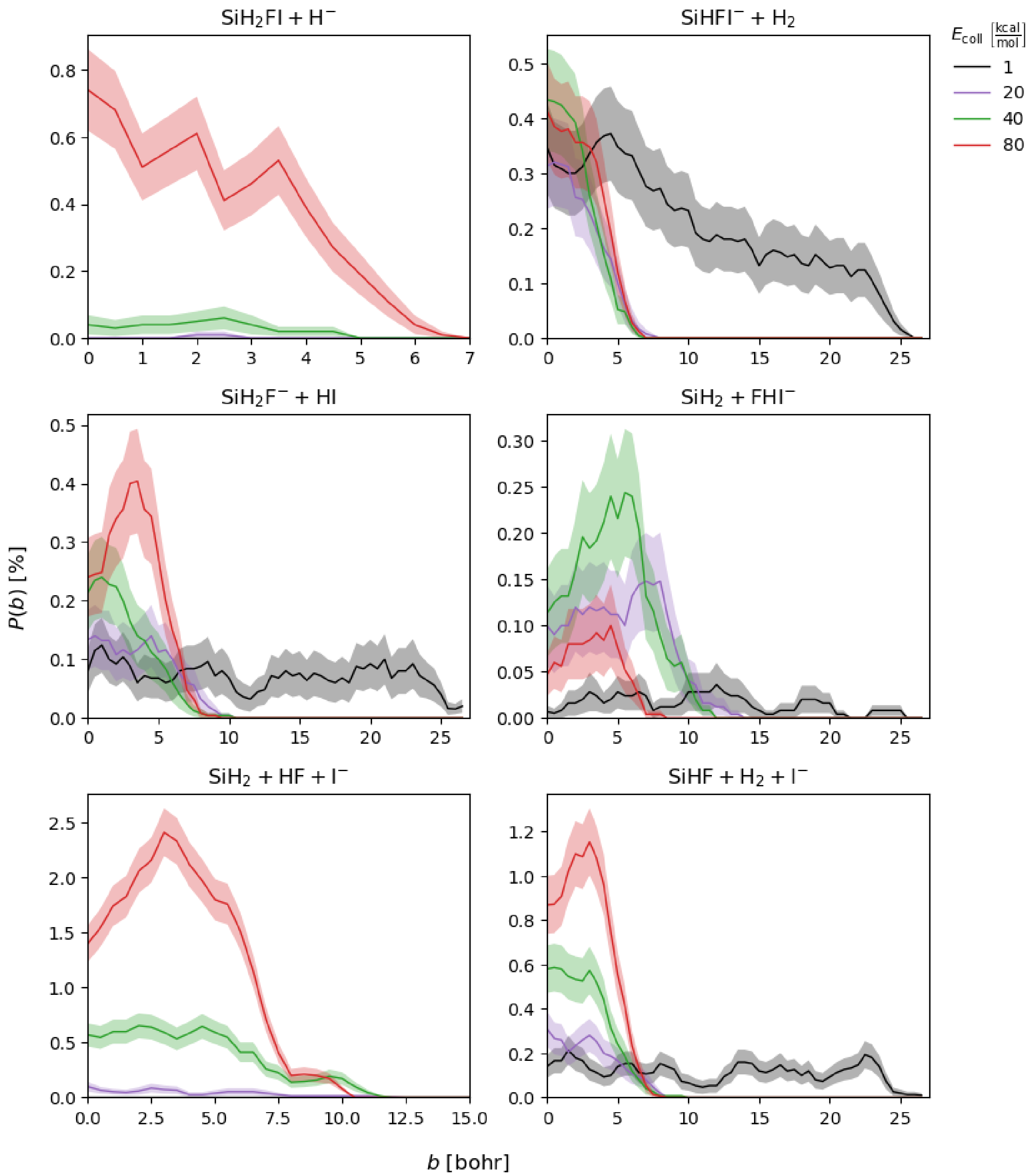

Figure S2. Reaction probabilities of the minor channels of the SiH_3I + F^- system as a function of impact parameter obtained at selected collision energies between E_{coll} = 1 and 80 kcal/mol. Shaded regions indicate ±1 standard error. Moving averages are used to represent the curves.

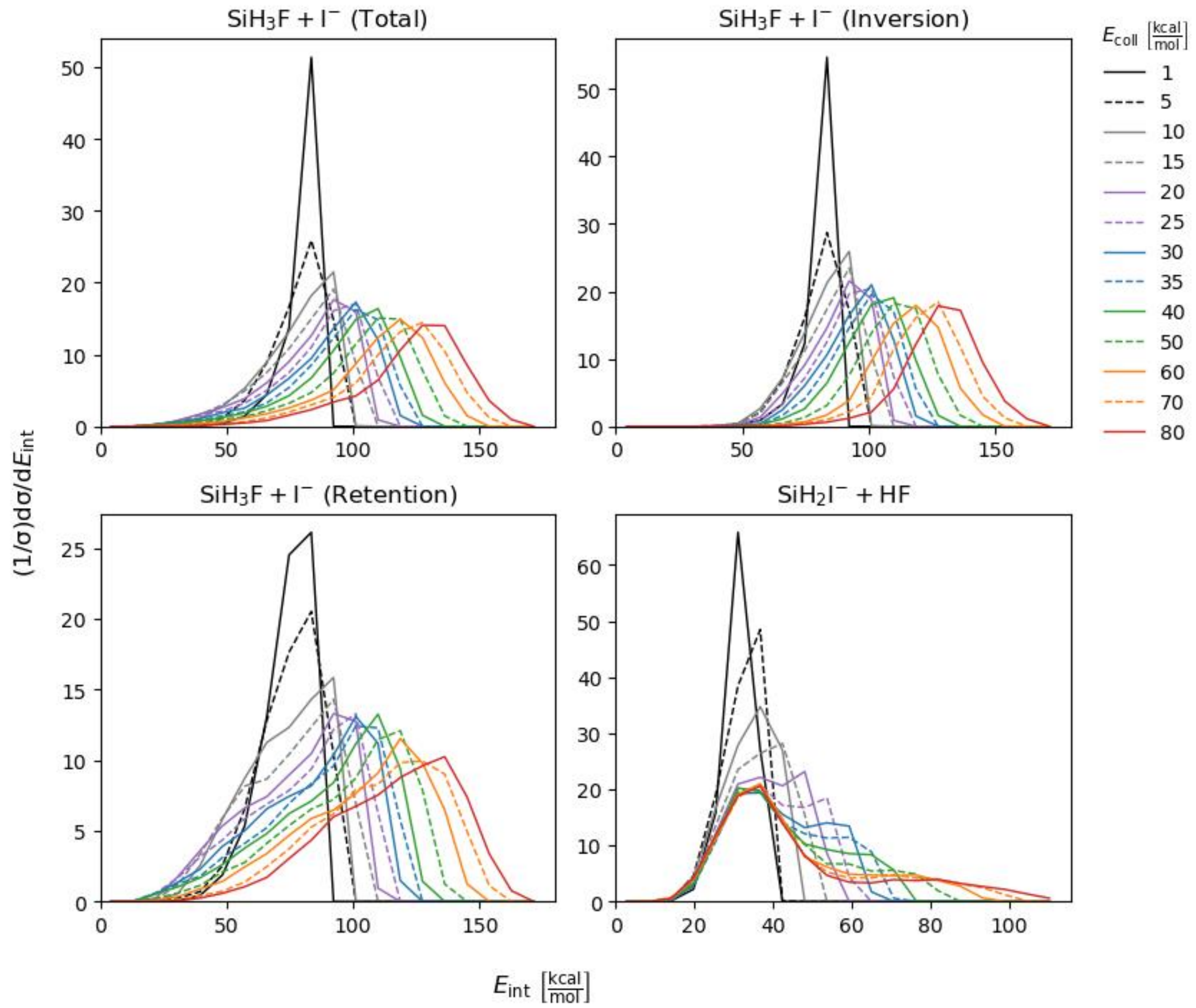

Figure S3. Normalized internal energy distributions of the major channels of the $SiH_3I + F^-$ system obtained at different collision energies between E_{coll} = 1 and 80 kcal/mol.

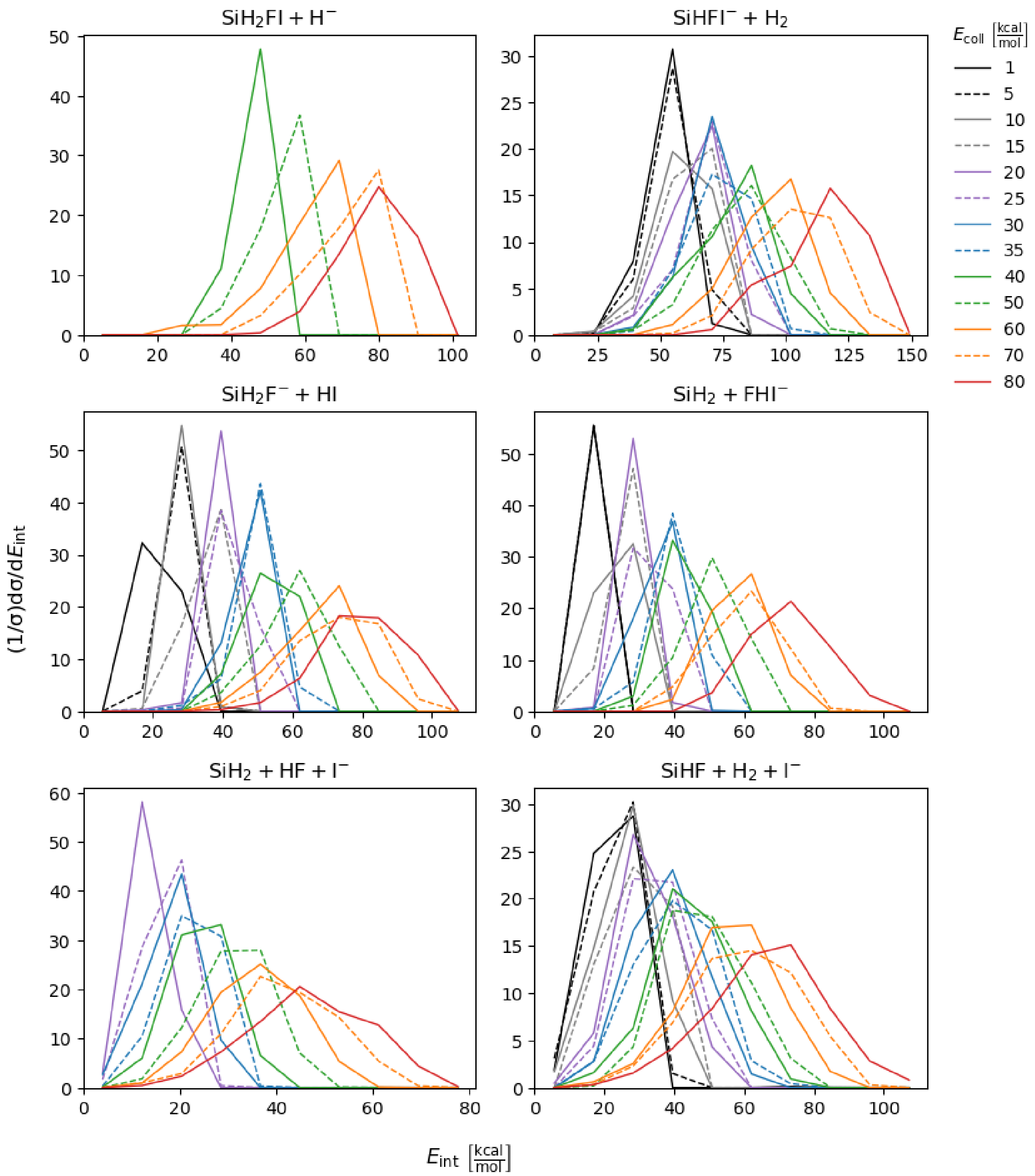

Figure S4. Normalized internal energy distributions of the minor channels of the $SiH_3I + F^-$ system obtained at different collision energies between E_{coll} = 1 and 80 kcal/mol.

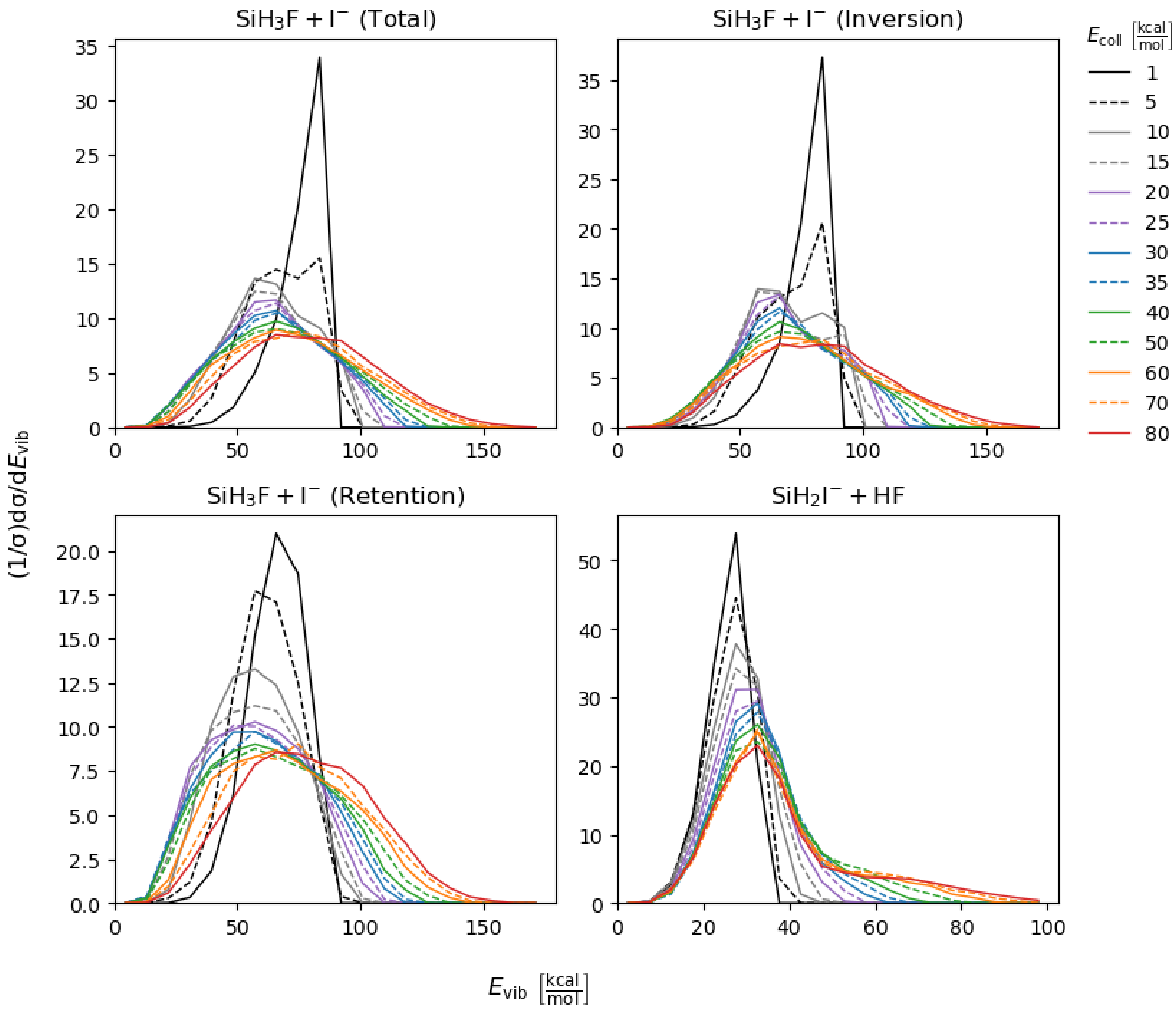

Figure S5. Normalized vibrational energy distributions of the major channels of the $SiH_3I + F^-$ system obtained at different collision energies between E_{coll} = 1 and 80 kcal/mol.

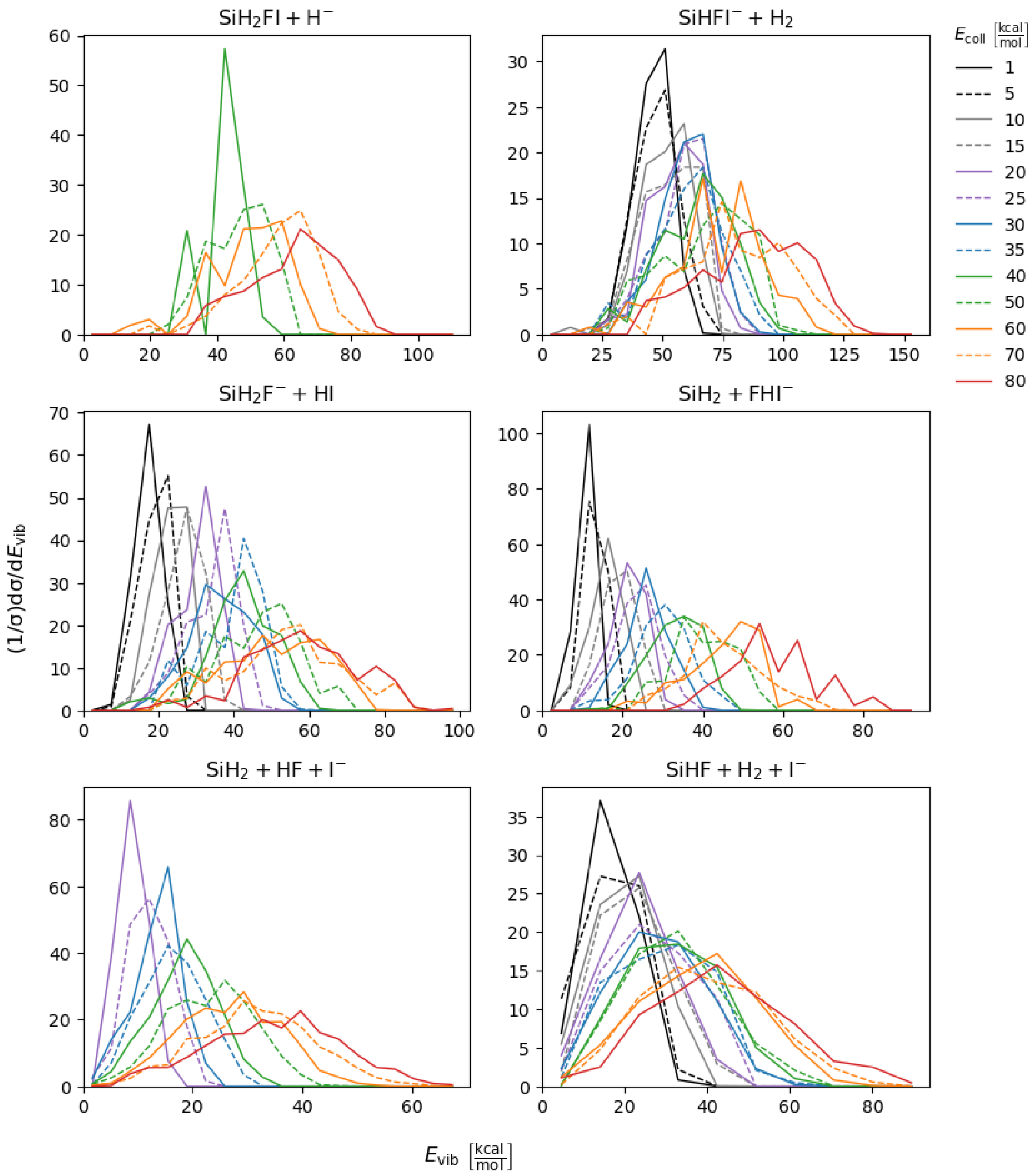

Figure S6. Normalized vibrational energy distributions of the minor channels of the $SiH_3I + F^-$ system obtained at different collision energies between E_{coll} = 1 and 80 kcal/mol.

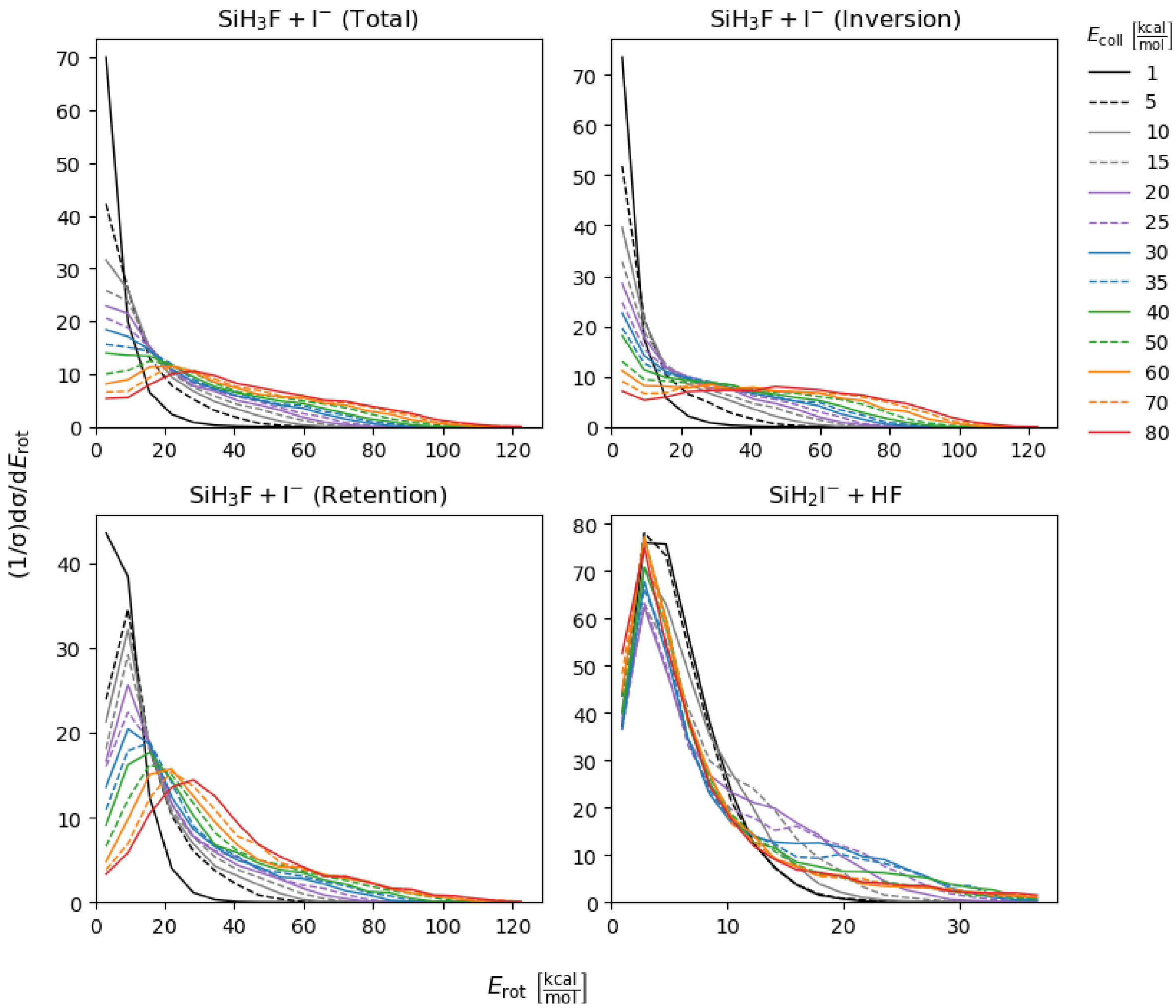

Figure S7. Normalized rotational energy distributions of the major channels of the $SiH_3I + F^-$ system obtained at different collision energies between E_{coll} = 1 and 80 kcal/mol.

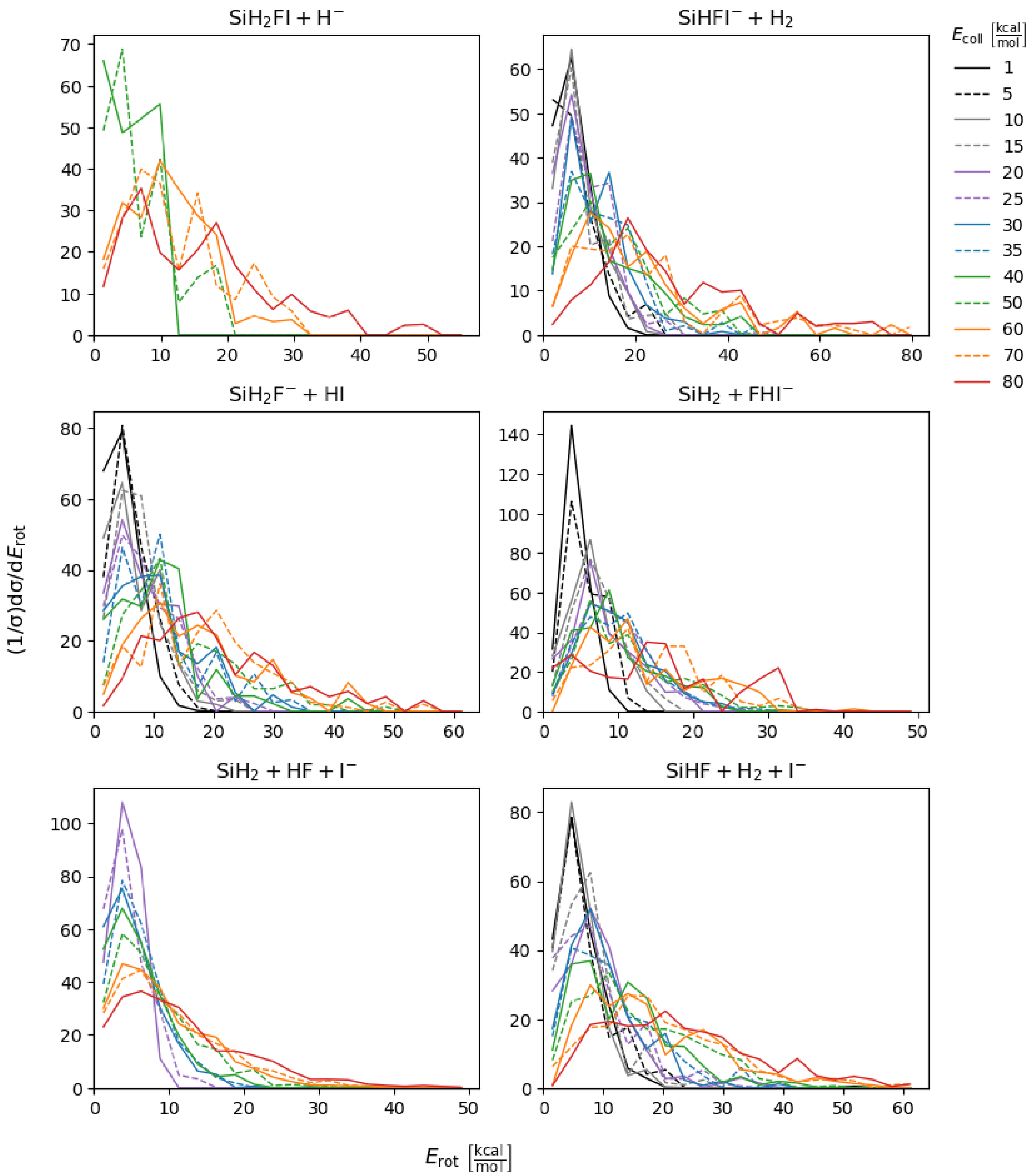

Figure S8. Normalized rotational energy distributions of the minor channels of the $SiH_3I + F^-$ system obtained at different collision energies between E_{coll} = 1 and 80 kcal/mol.

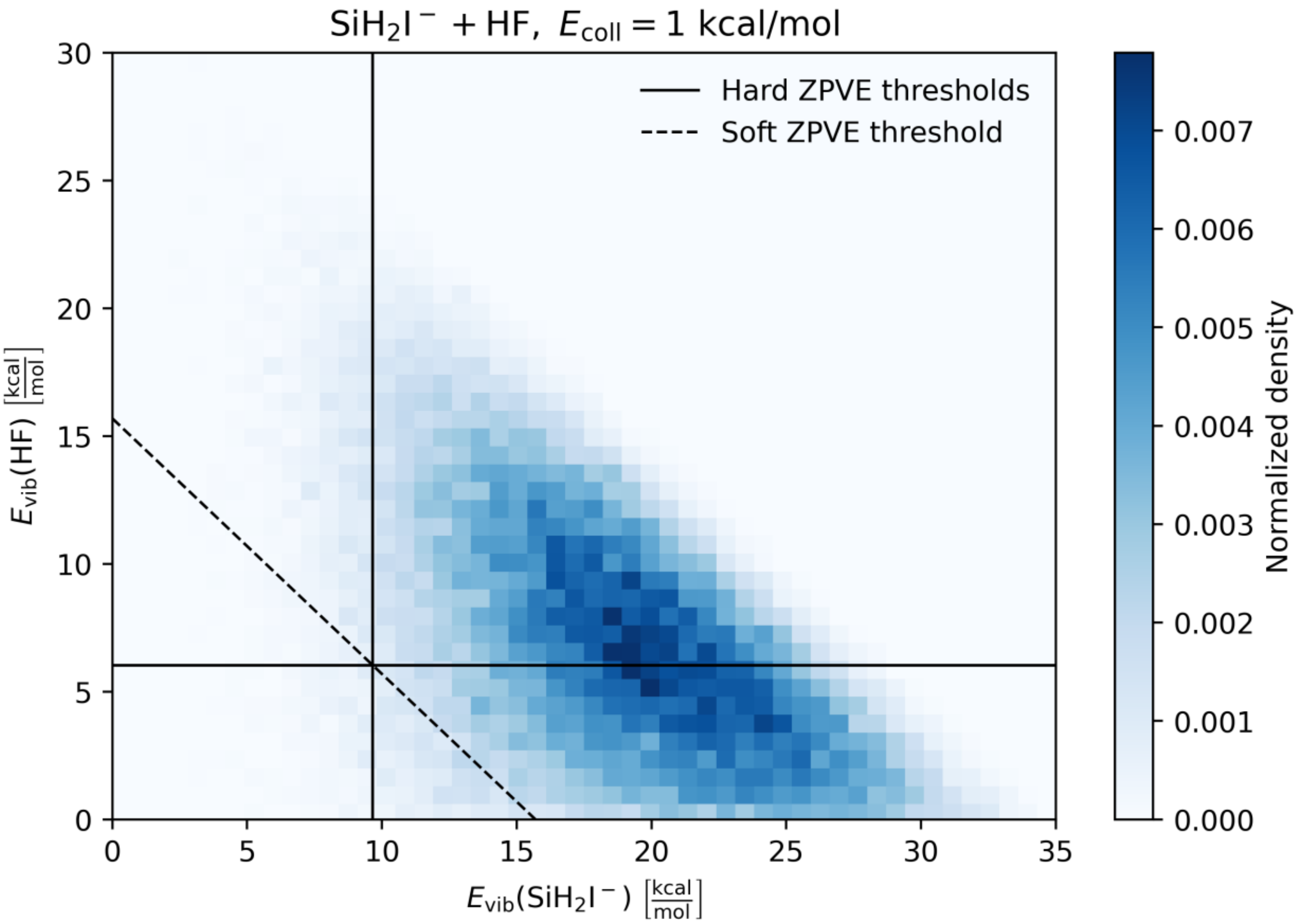

Figure S9. Normalized correlated vibrational-energy distribution of the SiH_2I^- + HF products at E_{coll} = 1 kcal/mol. The vertical and horizontal solid lines indicate the fragment-specific ZPVE thresholds used for the hard vibrational constraint, whereas the dashed diagonal line indicates the summed ZPVE threshold used for the soft constraint.

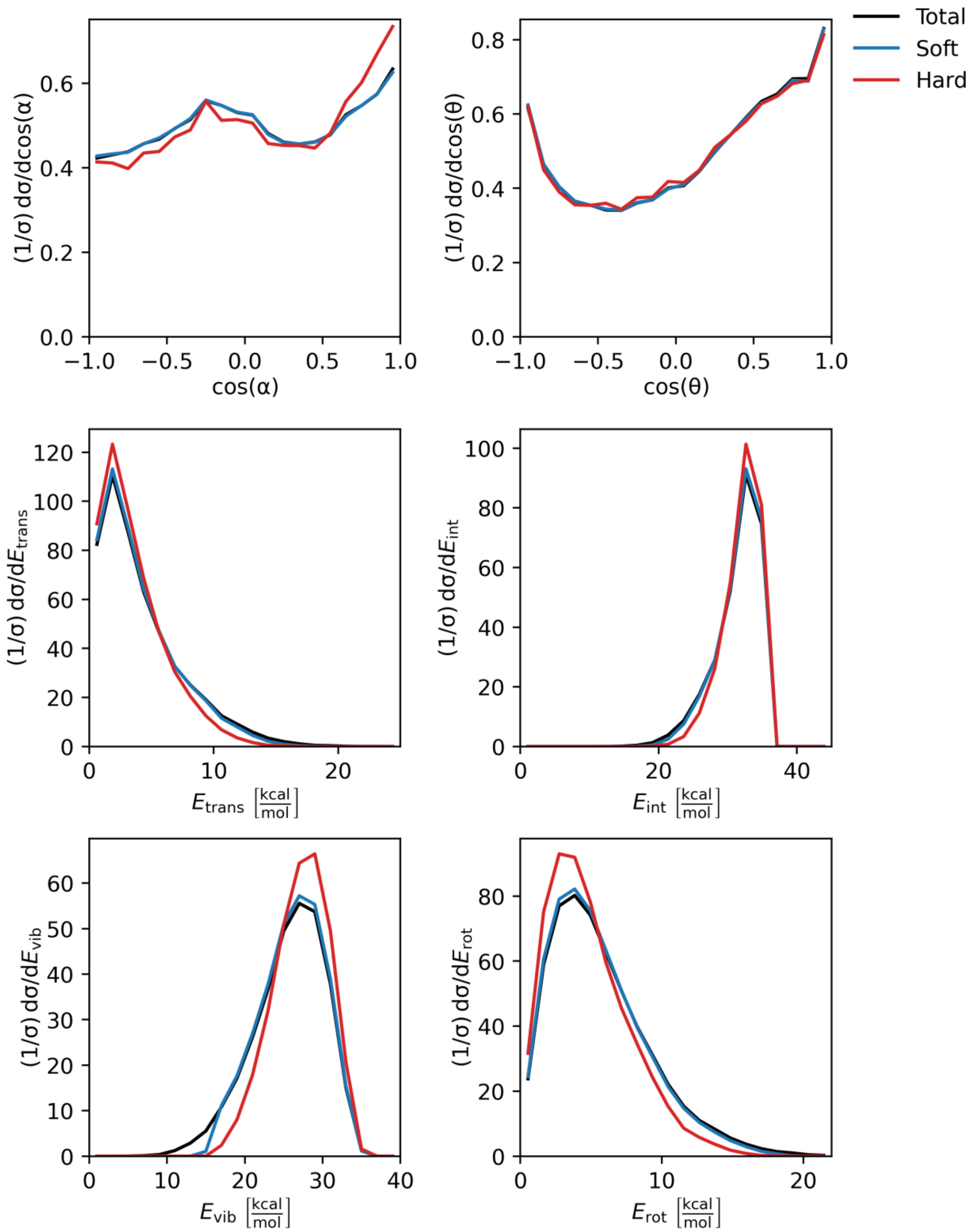

Figure S10. Normalized initial attack-angle, scattering-angle, and product translational, internal, vibrational, and rotational energy distributions for the SiH_2I^- + HF proton-abstraction channel at E_{coll} = 1 kcal/mol, obtained without vibrational constraints (Total) and with the soft and hard vibrational constraints.
